# Sandwich-Architected Hybrid Organic Crystals with Humidity–Temperature Sensing and Cryogenic Photothermal Actuation

**DOI:** 10.1007/s40820-025-01996-7

**Published:** 2026-01-05

**Authors:** Linfeng Lan, Lijie Wang, Chenguang Wang, Hongyu Zhang

**Affiliations:** 1 State Key Laboratory of Integrated Optoelectronics, College of Electronic Science and Engineering, Jilin University, Qianjin Street, Changchun, 130012 People’s Republic of China; 2 State Key Laboratory of Supramolecular Structure and Materials, College of Chemistry, Jilin University, Qianjin Street, Changchun, 130012 People’s Republic of China

**Keywords:** Organic crystals, Reduced graphene oxide composites, Humidity and temperature sensing, Cryogenic photothermal actuation

## Abstract

The growing demand for personalized health care, smart wearables, and advanced environmental monitoring has spurred the development of multifunctional materials that combine flexibility, environmental adaptability, and diverse functionalities. However, conventional materials often failed to integrate these attributes simultaneously, hindering their applicability in next-generation technologies. Here, we present an organic–inorganic hybrid crystalline material with a unique sandwich-like architecture, in which a flexible organic crystal core is encased by reduced graphene oxide (rGO) and thermoplastic polyurethane (TPU). This strategic integration endows the material with fluorescence, cryogenic flexibility, and electrical conductivity, while also enabling dual sensing and actuation capabilities. The rGO layer facilitates real-time humidity (25–90% RH) and temperature (25–180 °C) sensing through environmental interactions, whereas the differential thermal expansion between TPU and the flexible crystal core drives efficient photothermal actuation at − 150 °C for advanced thermal regulation. The hybrid material exhibits stable performance under extreme conditions, making it a promising candidate for biomedical monitoring, flexible electronics, and energy applications. This work establishes hybrid crystalline materials as versatile and scalable platforms for addressing complex technological demands, paving the way for their application in next-generation multifunctional devices.

**Supplementary Information:**

The online version contains supplementary material available at 10.1007/s40820-025-01996-7.

## Highlights

- A layered hybrid crystal integrates fluorescence, mechanical flexibility, conductivity, and cryogenic durability via reduced graphene oxide and thermally responsive polyurethane encapsulation.
- The hybrid crystal enables real-time dual-mode sensing of humidity (1.65% RH^−1^) and temperature (0.46% °C^−1^) with high sensitivity and cycling stability.
- Infrared-induced photothermal actuation at − 150 °C allows reversible crawling and walking under cryogenic conditions.

**Supplementary Information:**

The online version contains supplementary material available at 10.1007/s40820-025-01996-7.

## Graphical abstract

**Figure:**
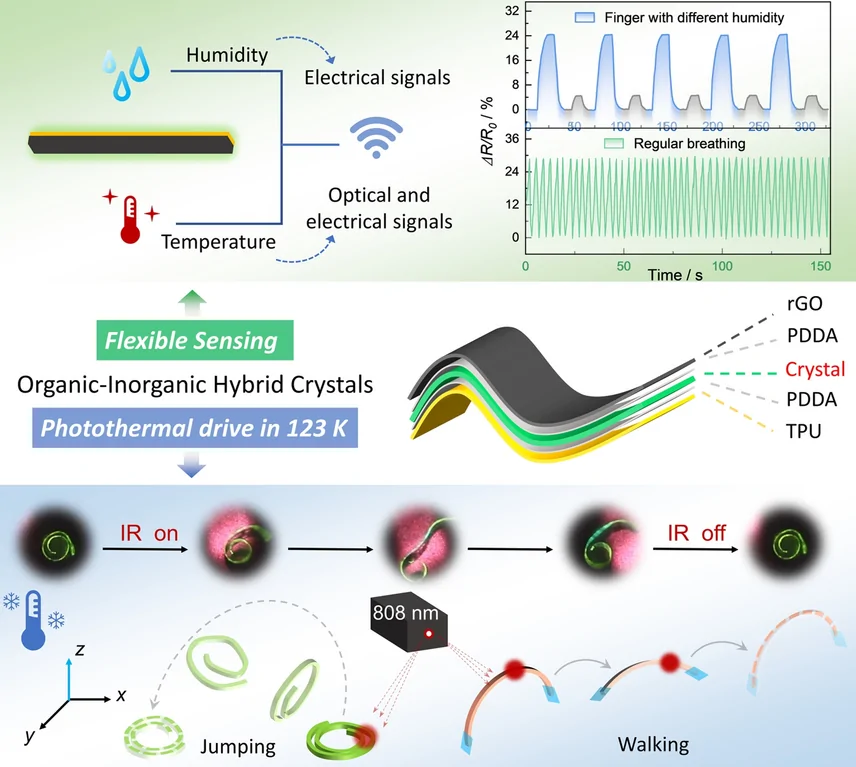

## Introduction

The advent of wearable flexible electronics marks a transformative leap in technology, with profound implications for diverse sectors, including health care, human–machine interfaces, environmental sensing, and next-generation communication systems [1, 2]. Unlike conventional rigid electronic devices, flexible electronics seamlessly conform to the human body, enabling continuous and unobtrusive monitoring of physiological and environmental parameters [3, 4]. A critical driver of this technological evolution is the development of multifunctional optoelectronic materials capable of addressing multifaceted demands of modern electronic applications. Essential characteristics of these materials include mechanical flexibility, tunable optoelectronic properties, and responsiveness to external stimuli such as temperature, light, and humidity [5–7]. Despite notable progress in material science, traditional materials such as metals, ceramics, and polymers often fall short in achieving the required balance of durability, reliability, and performance optimization under diverse conditions [8]. This limitation underscores the need for innovative material systems capable of synergistically integrating multiple functions within a single platform. Hybrid and composite materials [9] have emerged as promising candidates, as they enable the combination of mechanical [10], electrical [11], optical [12], and thermal characteristics [13] to create multifunctional systems with enhanced performance. For instance, integrating precious metal nanoparticles like gold and silver with two-dimensional graphene significantly enhances multifunctional properties of the latter, paving the way for advancements in fields such as sensing, photothermal therapy, and drug delivery [14].

Achieving true multifunctionality in materials is a significant challenge, necessitating a holistic approach that balances individual performance with component compatibility, integrating insights from materials chemistry, physics, and engineering. Within this framework, flexible organic crystals have attracted attention for their exceptional mechanical flexibility and outstanding optoelectronic properties [15–20]. Their inherent characteristics, including elasticity [21, 22], optical waveguides [23, 24], and environmental sensitivity [25, 26], position them as ideal candidates for applications [27–30] that require lightweight, adaptable, and responsive materials. Building on these principles, hybrid crystalline materials have emerged as promising solutions, offering tunable structures and multifunctional properties [31–35]. Recent progress in sandwich-structured and organic–inorganic integrated sensing systems demonstrated that rational structural design enables multimodal sensing and intelligent control, as exemplified by mechanoluminescent–triboelectric bimodal sensors [36], vector piezoelectric sensors with anisotropic recognition [37], bioinspired electronic skins [38] and AI-assisted mechanoluminescent platforms [39]. These advances underscore the potential of hybrid architectures in flexible electronics and set the context for crystalline hybrid system as a novel approach. In this work, we develop a hybrid fluorescent crystalline material with a distinctive layered architecture, where reduced graphene oxide (rGO) and thermally responsive polyurethane (TPU) encapsulate a flexible organic crystal core. The crystal endows the composite material with optical transmission capabilities and cryogenic flexibility, while the rGO component provides electrical conductivity, temperature and humidity sensitivity, and a pronounced photothermal effect. Additionally, the thermal expansion mismatch between TPU and the crystal core serves as a driving force for temperature-induced actuation in cryogenic environments. This innovative configuration integrates multiple functionalities into a single material platform, enabling precise humidity and temperature sensing with sensitivities of 1.65% RH^−1^ and 0.46% °C^−1^, respectively. Furthermore, the synergistic interplay between the photothermal effect of rGO and the cryogenic adaptability of the polymer–crystal system facilitates precise photothermal actuation, representing a rare example of controllable crystal motion at cryogenic temperatures (down to − 150 °C), including crawling, obstacle crossing, and walking. This advancement presents exciting opportunities for remote-controlled actuation in extreme environments. Unlike liquid crystal elastomers or photoresponsive smart materials [40–44], our hybrid crystals combine cryogenic photothermal actuation with dual-mode sensing in a multilayer architecture, offering functions beyond prior systems. By leveraging a holistic materials design strategy, this work highlights hybrid crystalline materials as versatile platforms that can address the complex demands of next-generation multifunctional devices. The integration of tunable structural and functional characteristics underscores their transformative potential in applications ranging from flexible electronics to advanced sensing technologies.

## Experimental Section

### Material Preparation

The materials for organic syntheses were obtained from commercial sources and were used as received. (*Z*)-4-(2-cyano-2-(4-(trifluoromethoxy)phenyl)vinyl)benzonitrile (compound **1**), (*E*)-4-(3-(5-methoxy-1-methyl-1*H*-indol-3-yl)acryloyl)benzonitrile (compound **3**), and (Z)-3-(furan-2-yl)-2-(4-(((E)-2-hydroxy-5-methylbenzylidene)amino)phenyl)acrylonitrile (compound** 4**) were synthesized according to procedures described previously (Scheme S1) [32, 45, 46]. 2, 2ʹ-((1*E*, 1ʹ*E*)-1, 4-phenylenebis(ethene-2, 1-diyl))dibenzonitrile (compound **2**) was purchased from Energy Chemical (Beijing, China) and further purified by column chromatography using dichloromethane and petroleum ether (V/V = 2:1) as the eluent. Poly(diallyldimethylammonium chloride) (PDDA; average Mw: 200,000–350,000; 20 wt% in H_2_O) solution was purchased from Sigma-Aldrich. Reduced graphene oxide (purity: > 99 wt%; thickness: ~ 2 nm; layer diameter: 0.2 ~ 10 μm; number of layers: 1 ~ 2; and specific surface area: 100 ~ 500 m^2^ g^−1^) was purchased from Suzhou Tanfeng Graphene Technology (Suzhou, China). Thermoplastic polyurethane (Elastollan 1195A TPU) was purchased from BASF (Germany).

### Crystal Growth

To prepare the crystal **1**, approximately 100 mL of a solution of compound **1** in dichloromethane (DCM; ~ 5 mmol L^−1^) was added to a 500-mL flask, and an approximately identical volume of petroleum ether was carefully added along the wall. After allowing for a slow diffusion by keeping the crystallization mixtures at 5 °C for two days, high-quality needle-shaped crystals **1** were obtained. By adding ethanol to dilute solutions of **2**–**4** (dissolved in DCM at concentrations of ~ 8, 10, and 15 mmol L^−1^ for **2**, **3**, and **4**, respectively), crystals **2**–**4** were obtained after the crystallization mixtures were kept at room temperature for 3–5 days.

### Preparation of Hybrid Crystals

Crystals **1**–**4** were immersed in an aqueous solution of PDDA containing 5.0 mg mL^−1^ PDDA and 1.0 M NaCl for 20 min, followed by gentle drying with an N_2_ flow. The rGO was dispersed in an aqueous solution to ensure a stable suspension and effective interaction with the PDDA-coated crystal. The crystals were then fixed onto a glass substrate, and an aqueous dispersion of reduced graphene oxide (rGO, 1 mg mL^−1^) was drop-cast onto their surface. After the water evaporated naturally, the crystals underwent thermal annealing at 60 °C for 30 mins. The commercial TPU dissolved in tetrahydrofuran facilitates the rapid diffusion and evaporation of the solvent, resulting in a uniform coating. Finally, a thin film of TPU ethanol solution was applied to the opposite side of the rGO-coated crystals. After solvent evaporation, the organic–inorganic hybrid crystals rGO/PDDA/Cry/PDDA/TPU (denoted as GT**1**–**4**) were obtained. In this study, rGO was chosen for its stability in humid environments and facile solution processability, which facilitates the reproducible fabrication of robust hybrid crystal sensors. Among the various polymers explored in our previous studies, TPU was selected for its mechanical flexibility, reproducibility, and ease of processing [31], which support reliable hybrid crystal assembly. In addition, reference samples Cry/PDDA, TPU/PDDA, rGO/PDDA, and Cry/PDDA/TPU were also prepared using the same procedure.

### Characterization

The UV − Vis absorption spectra were recorded with a Shimadzu UV-2550 spectrophotometer. Scanning electron microscopy (SEM) photographs and energy-dispersive *X*-ray spectroscopy (EDX) analysis were obtained using an FEI Quanta 450 environmental scanning electron microscope (ESEM) and a SU8020/Regulus8100 cold field emission scanning electron microscope (FSEM), respectively, both operated at 5–10 kV. *X*-ray photoelectron spectroscopy (XPS) measurements were carried out using a Shimadzu AXIS SUPRA + *X*-ray photoelectron spectrometer. The surface texture and roughness data were obtained on a BRUKER ICON-XR atomic force microscope (AFM). Optical photographs of crystals were obtained using a Canon camera or an Olympus BX61 optical microscope. The three-point bending and tensile tests were conducted using an Instron 5944 universal testing system, equipped with either a 5 or 10 N capacity and an Instron 2530 load cell. Differential scanning calorimetric (DSC) measurements were taken on a TA DSC Q20 instrument. Thermogravimetric analysis (TGA) was performed on a Waters Corporation TGA 550 instrument under a nitrogen atmosphere with a heating rate of 10 °C min^−1^. Contact angles were measured using a KRUSS DSA 30 contact angle system. For the optical waveguiding tests, the crystal was irradiated by the third harmonic (355 nm) of a Nd:YAG (yttrium–aluminum–garnet) laser with a pulse duration of about 5 ns. The energy of the laser was adjusted by using calibrated neutral density filters. The beam was focused onto a stripe whose shape was adjusted to 0.5 × 0.5 mm^2^ by using a plano-convex lens and a slit. The crystal was placed on a silicon wafer, and one tip of the crystal extended out of the edge of the silicon wafer to align with the probe of the spectrometer. While changing the irradiation locations, spectral data were collected for each irradiated location at the excitation site and at the tip of the crystal. All emission spectra were recorded on a Maya2000 Pro CCD spectrometer. The optical loss coefficients (*α*) were determined by single-exponential fitting of the function *I*_tip_/*I*_body_ = *A*exp(–α*D*) [28], in which *I*_tip_ and *I*_body_ are the fluorescence intensities of outcoupled and incident light, respectively, *A* is the optical loss coefficient, and *D* is the distance between the excited site and the tip of the crystals for collecting emission. The current signals were collected by a Keithley 2400 source meter. Humidity under different conditions was measured by a SongJing hygrometer. Low temperatures were measured by a Miaoxin T10R-PT temperature recorder. Infrared imaging photographs and movies were obtained from a Hikmicro HM-TPK20-3AQF/W handheld thermograph. 808-nm infrared light was obtained from a MXL-III-808 infrared semiconductor laser.

### Humidity-Sensing Performance Measurements

It should be noted that GT**2** represents a prototype hybrid crystal system rather than a fully optimized device; the reported data reflect typical responses across multiple independently prepared samples; and further development is required to realize fully functional devices. The performance of a humidity sensor can be evaluated through several key parameters, including sensitivity, operating range, response time, and stability. Among those, higher sensitivity and low detection limit are considered the most critical defining factors of a humidity sensor in high-reliability circuits and systems. The normalized resistance change (*ΔR/R*_*0*_; *ΔR* = *R* –* R*_*0*_), sensitivity (|*S*|), and method detection limit (*MDL*) of the resistive humidity sensor are defined as follows [47]:

1 $${\mathrm{Normalized}} \;{\mathrm{resistance}} \;{\mathrm{change}}\,[\% ] = (R - R_{0} ) / R_{0} \times 100$$

where *R* represents the real-time measured resistance and *R*_0_ is the initial resistance of the sensor at the reference relative humidity (25% RH) and at a temperature of 25 or 37 °C.

2 $${\mathrm{Sensitivity}}\,[\% \mathrm{RH}^{-1}] = |R - R_{0} | /(R_{0} \times \Delta {\mathrm{RH}}) \times 100 = |S|$$

where *Δ*RH is the relative humidity variation and *S* is the slope of the calibration curve. Sensitivity (% RH^−1^) is defined as the normalized resistance change rate induced by a unit variation in relative humidity (1% RH), reflecting the sensor’s responsiveness to humidity changes.

3 $${\text{MDL [\% RH]=}}{\times}K\,{\times}\,{\overline{\mathrm{SD}}}\, {/|}S{|}$$

where *K* is a numerical factor (3) chosen according to the confidence level desired and $\overline{\mathrm{SD}}$ (%) is the average of the standard deviation. *MDL* (% RH) is defined as the minimum humidity change that the sensor can reliably detect, which is related to the noise level and sensitivity.

### Temperature-Sensing Performance Measurements

Similar to humidity-sensing tests, the normalized resistance change (*ΔR/R*_*0*_), sensitivity and method detection limit (*MDL*) of the resistive temperature sensor are defined as follows:

4 $${\text{Normalized }}\;{\mathrm{resistance}}\;{\mathrm{change}}\,[\% ] = (R{-}R_{0} )/R_{0} \times 100$$

where *R* represents the real-time measured resistance and *R*_0_ is the initial resistance of the sensor at the reference temperature (25 °C) and at a humidity of 30% or 90% RH. Sensitivity (% °C^−1^) is defined as the normalized resistance change rate induced by a unit temperature variation (1 °C), reflecting the sensor’s responsiveness to temperature changes.

5 $${\mathrm{Sensitivity}}\,[\% ^\circ {\mathrm{C}}^{-1}] = |R{-} R_{0} | /(R_{0} \times \Delta T) \times 100 = |S|$$

where *ΔT* is the temperature variation and *S* is the slope of the calibration curve.

6 $${\mathrm{MDL}}\,[^\circ {\mathrm{C}}] = K{\times}{\overline{\mathrm{SD}}} /|S|$$

where *K* is a numerical factor (3) chosen according to the confidence level desired and ${\overline{\mathrm{SD}}}$ (%) is the average of the standard deviation. *MDL* (°C) is defined as the minimum temperature change that the sensor can reliably detect, which is related to the noise level and sensitivity.

### Human Monitoring Tests

Informed signed consent was obtained from the volunteers who participated in the human monitoring test. With the voltage through the hybrid crystal sensor maintained at 1 V, a human index finger was either repeatedly passing over the hybrid crystal or positioned at varying distances directly above it. Time-resolved current/resistance response curves were recorded. The sensor was also attached to other body parts, including below the nose, to capture time-dependent current/resistance changes under different conditions.

### Controlled Motion and Signal Output at Low Temperatures

The prepared hybrid crystals GT**2**–**4** were placed on the surface of a silicon wafer, which was subsequently immersed in liquid nitrogen. Under these conditions, the initially straight crystals immediately bent or curled, with the degree of deformation depending on the thickness of crystals **2**–**4** [31]. A power-adjustable 808 nm near-infrared (NIR) beam (0–250 mW) was used to irradiate specific regions of the crystals. The rapid local temperature rise induced asymmetric deformation of the curled crystals at low temperatures, resulting in controlled motion. When the crystal ends were fixed and connected to a source meter via metallic probes, repeated infrared irradiation caused the crystals to deform and recover cyclically, enabling the generation of periodic signal outputs.

## Results and Discussion

### Fabrication and Structural Characterizations

**Fig. 1:**
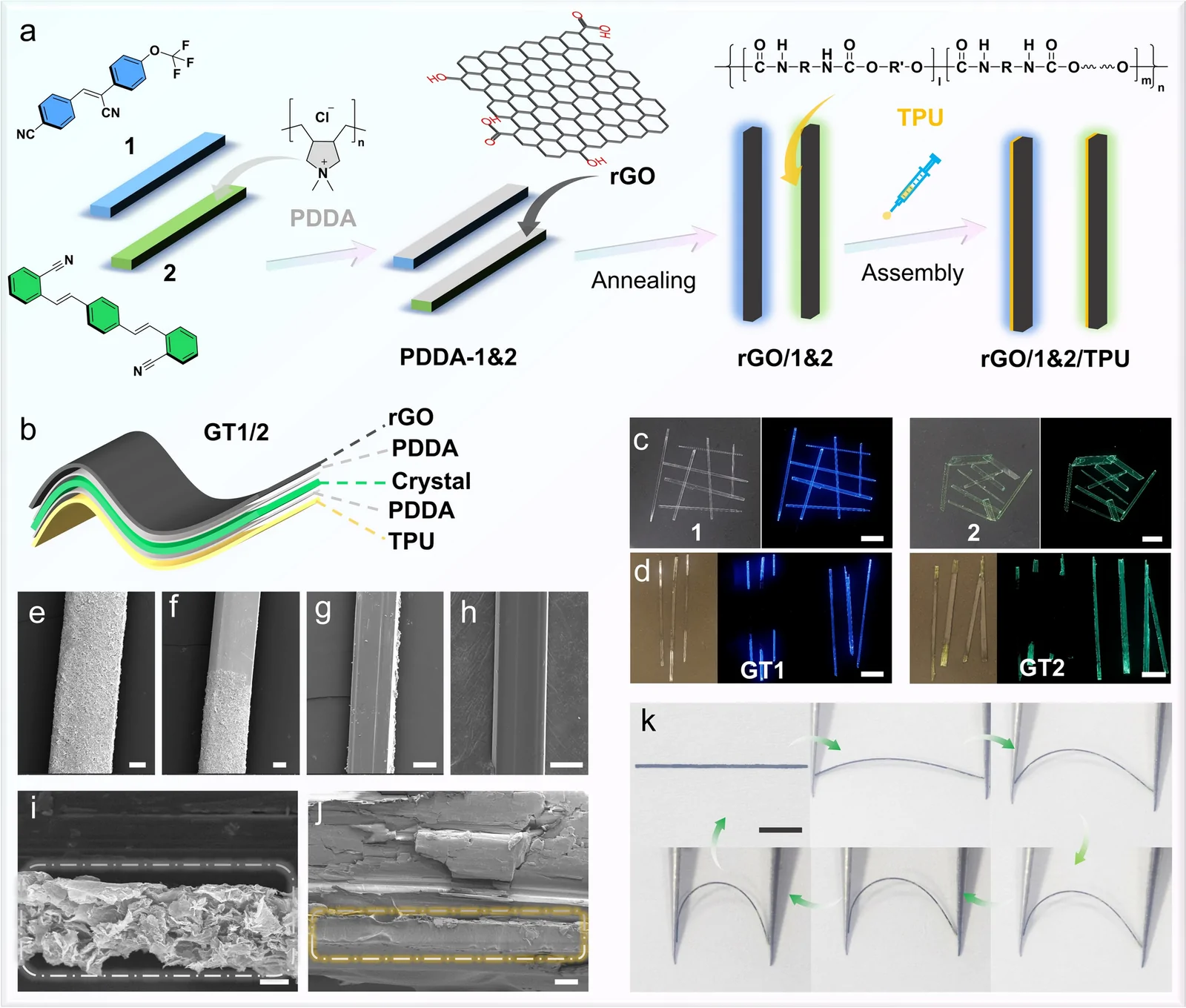
Preparation of the sandwich structure hybrid crystal. Schematic of **a** the preparation process and **b** sandwich multilayer structure of hybrid material GT**1**, GT**2**. Blue and green represent the crystal layer, gray represents the PDDA layer, black represents the rGO layer, and yellow represents the polyurethane layer.** c** Photograph of original crystals **1** and **2** under daylight and 365-nm UV light.** d** Photograph of hybrid crystals GT**1**, GT**2** showing both sides of the curved plane. Scanning electron micrographs showing **e** the rGO layer, **f** interface between the rGO layer and the crystal, **g** backside of the rGO-modified crystal, and **h** TPU layer. Scanning electron micrographs showing a cross section of **i** rGO and **j** TPU layer. The gray and yellow dashed lines indicate the rGO and TPU layer, respectively.** k** Reversible bending deformation of GT**2**. Scale bars: 1 mm in panels (**c**, **d**); 100 μm in panels (**e**–**g**); 50 μm in panel (**h**); 5 μm in panel (**i**); 10 μm in panel (**j**); 5 mm in panel (**k**)

Compounds **1**–**4** were synthesized following reported procedures and crystallized via solvent diffusion [32, 45, 46]. Crystals **1** and **2**, which yield sheetlike and needlelike structures with centimeter-scale lengths, serve as the primary focus of this study (Fig. 1a, c). Crystals **3** and **4**, which grow into thin needlelike crystals (10–20 μm in thickness), are particularly suitable for photothermal actuation (discussed in detail in Sect. 3.4). These crystals were chosen as substrates due to their mechanical flexibility, high fluorescence quantum efficiency, and cost-effectiveness, making them ideal candidates for constructing multilayer architectures and facilitating comprehensive functional studies. To construct the hybrid crystals, a self-assembled layer of poly(diallyldimethylammonium chloride) (PDDA) was first deposited onto the crystal surface, imparting a positive charge to enable uniform adsorption of reduced graphene oxide (rGO) [48]. Subsequently, layers of rGO and TPU were sequentially deposited onto the wide surfaces of the crystals, producing multilayer hybrid crystals rGO/PDDA/Crystal/PDDA/TPU (Fig. 1a, b), designated as GT**1** and GT**2**, where “G” and “T” indicate the rGO and TPU layers, respectively, and the numbers **1** and **2** refer to crystals **1** and **2**. Energy-dispersive *X*-ray spectroscopy (EDX) confirms the presence and uniform distribution of the key elements in GT**1**, verifying the successful assembly of the layers (Fig. S1 online). *X*-ray photoelectron spectroscopy (XPS) further reveals interfacial interactions between the different layers rather than mere physical stacking (Fig. S2). Specifically, in **2**/PDDA, the C–N component downshifts by ~ 0.2 eV compared with pristine crystal **2**; in rGO/PDDA, the O 1*s* components shift by 0.3–0.7 eV with changes in relative intensity; and TPU/PDDA shows a ~ 0.3 eV downshift of the C–O/C–N component, accompanied by variation in the carbonyl peak intensity. The observed binding energy shifts reveal the presence of interactions between the layers, which help stabilize the multilayer structure.

**Fig. 2:**
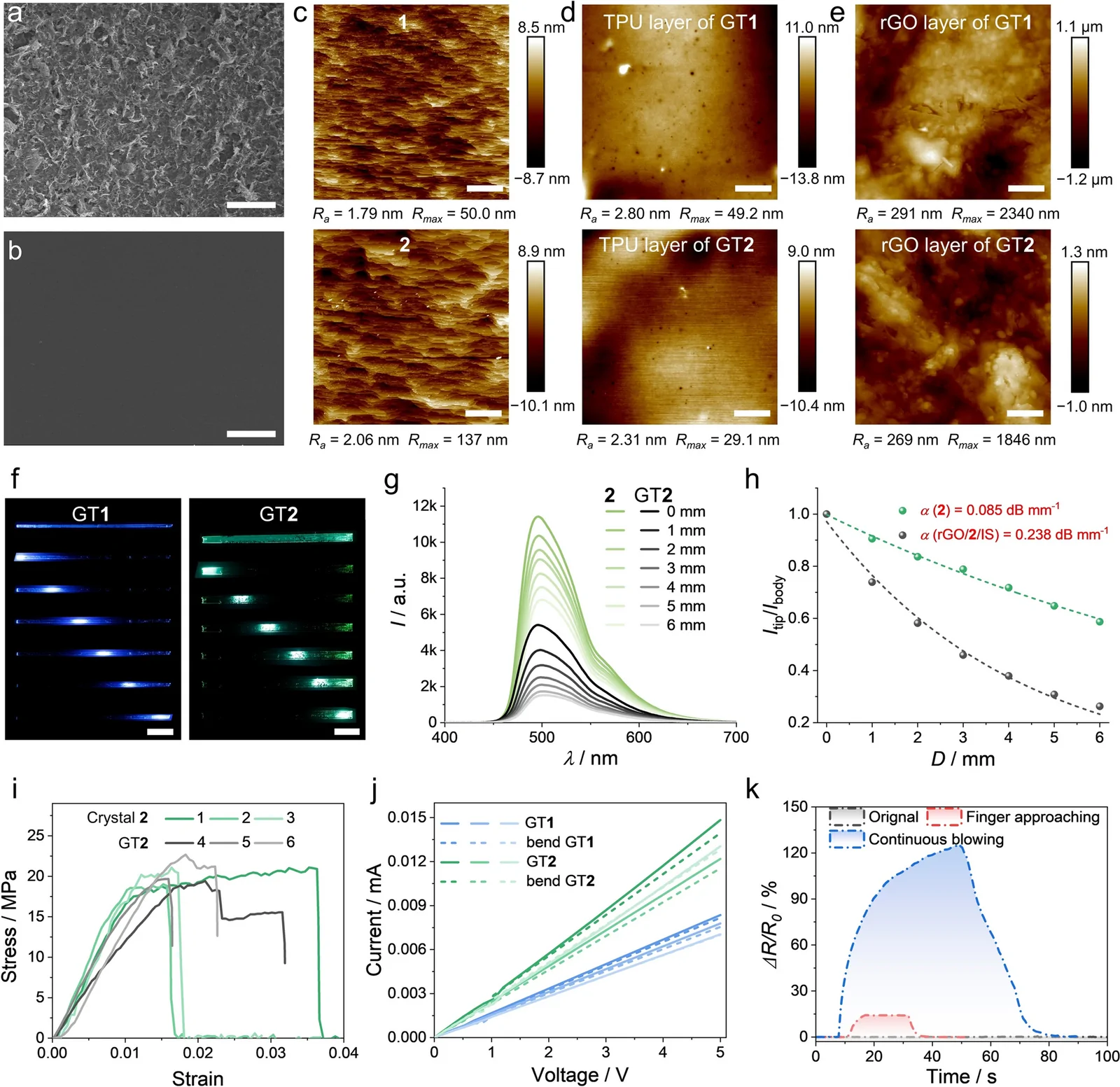
Surface morphology and physical properties of GT**1** and GT**2**. The surface morphologies of the GT**2** crystal on both sides including **a** rGO and **b** TPU layers after 1000 bending–recovery cycles.** c** AFM images of crystals **1** and **2**, and surface morphology of GT**1** and GT**2**, showing **d** TPU layer and **e** rGO layer.** f** Fluorescent images and **g** emission spectra of GT**1** and GT**2** collected at different distances between the excitation site and the detection tip.** h** *I*_tip_/*I*_body_ decays of GT**2**.** i** Stress–strain curves obtained from the three-point bending tests of crystals **2** and GT**2**.** j** Current–voltage (*I*–*V*) curves of different hybrid crystals in straight and bent states.** k** Normalized resistance change (*ΔR/R*_*0*_; *ΔR* = *R* –* R*_*0*_) over time for GT**2** under ambient conditions, finger proximity, and air-blowing conditions. Scale bars: 30 μm in panels (**a**, **b**); 3 μm in panels (**c**–**e**); 1 mm in panel (**f**)

Additionally, hybrid crystals featuring rGO deposited exclusively in their central regions were fabricated to enhance structural observation (Fig. 1d). Upon excitation with a 365 nm ultraviolet (UV) light, the rGO-modified regions exhibited negligible luminescence due to the inherent black opacity of rGO, whereas the transparent TPU caused minimal interference, preserving the inherent fluorescence of crystals. Scanning electron microscopy (SEM) confirmed the dense and uniform distribution of rGO on the surface of the crystal (Figs. 1e, f and S3), while the opposite side retained its pristine crystalline morphology (Fig. 1g). Subsequent deposition of the polymer layer maintained a smooth and flat surface (Fig. 1h). Cross-sectional analyses revealed tightly adhered and homogeneously distributed rGO and TPU layers, with thicknesses of 13.47 ± 0.93 and 11.07 ± 0.43 μm, respectively (Figs. 1i, j and S4). SEM and atomic force microscopy (AFM) analyses further confirmed the integrity of the multilayer hybrid crystals and the uniform deposition of the thin PDDA layer on the crystal surface (Figs. S5a, c and S6a, b), providing direct evidence of the intended sandwich-like structure. Macroscopic mechanical testing demonstrated that both the pristine crystal **2** and hybrid crystal GT**2** could be reversibly bent using tweezers without fracture (Figs. 1k and S7, S8). Even after 1000 bending and recovery cycles, the rGO layer on GT**2** maintained its uniform lamellar structure, and the TPU layer remained intact, demonstrating excellent interlayer adhesion and structural stability (Figs. 2a, b and S9).

### Optical, Electrical, and Mechanical Properties of Hybrid Crystals

AFM images further revealed a significant increase in the surface roughness of the rGO layers in hybrid crystals compared to pristine crystals, with the average roughness (*Ra*) increasing from 1.79 to 291 nm (GT**1**) and from 2.06 to 269 nm (GT**2**), while the TPU layers retained similar roughness levels to the original crystals (Fig. 2c–e). The distinct structural features of the hybrid crystals provided an ideal basis for a rigorous evaluation of their optical waveguiding and mechanical performance. When GT**1** or GT**2** with the TPU layer facing upward was excited by a 355 nm laser, blue or green emissions were transmitted from one end to the other (Fig. 2f). The strong absorption of rGO reduced the output signal intensity of GT**1** and GT**2** to 47% ~ 57% of the original values, increasing the optical loss coefficients from 0.079 and 0.085 dB mm^–1^ to 0.219 and 0.238 dB mm^–1^, respectively (Figs. 2g, h and S10). These values remain relatively low compared to reported organic crystals [49], indicating that the hybrid crystals maintain excellent optical transmission. The mechanical properties of hybrid crystals were investigated by three-point bending and tensile tests. In the bending mode, the stress–strain curves of the pristine crystals **1** and **2** exhibit an initial linear elastic region followed by a plastic deformation plateau (Figs. 2i and S11, S12), with fracture strengths of 7–21 MPa, maximum elastic strains of ~ 1%, and elastic modulus of 1.91 ± 0.08 and 1.85 ± 0.10 GPa. (Table S1). After hybridization, the yield strengths of GT**1** and GT**2** remain comparable, maximum elastic strains slightly increase (> 1%), and elastic modulus decreases to 1.54 ± 0.11 and 1.35 ± 0.22 GPa, reflecting enhanced deformability due to the soft polymer layer accommodating additional strain under stress. In the tensile mode, each crystal was split into two segments for direct comparison. The averaged tensile moduli of crystal **1** (1.22 ± 0.13 GPa) and GT**1** (1.21 ± 0.11 GPa), as well as crystal **2** (0.66 ± 0.04 GPa) and GT**2** (0.69 ± 0.01 GPa), are essentially identical (Table S2). Fracture strength and maximum strains remain comparable, indicating that hybridization does not significantly affect mechanical performance under stretching (Fig. S13). Moreover, the hybrid crystals maintained stable electrical conductivity (~ 0.21 S cm^–1^) over multiple bending cycles, with the rGO layer serving as a continuous conductive network that enabled reliable charge transport under mechanical stress [50], as evidenced by only a slight increase in resistance after repeated bending (Fig. 2j). In addition to their optical and electrical properties, the hybrid crystals demonstrated sensitivity to environmental humidity, with electrical signal changes observed when a human finger approached or air was blown onto the surface (Figs. 2k and S14). The rGO used here was prepared by chemical reduction and still retains a small amount of oxygen-containing functional groups, which endows it with hydrophilic behavior (Fig. S15) and enables effective interaction with moisture [51, 52]. Overall, these findings demonstrate that the hybrid crystals maintain consistent optical, electrical, and mechanical properties even under cyclic deformation.

### Humidity- and Temperature-Sensing Performance

**Fig. 3:**
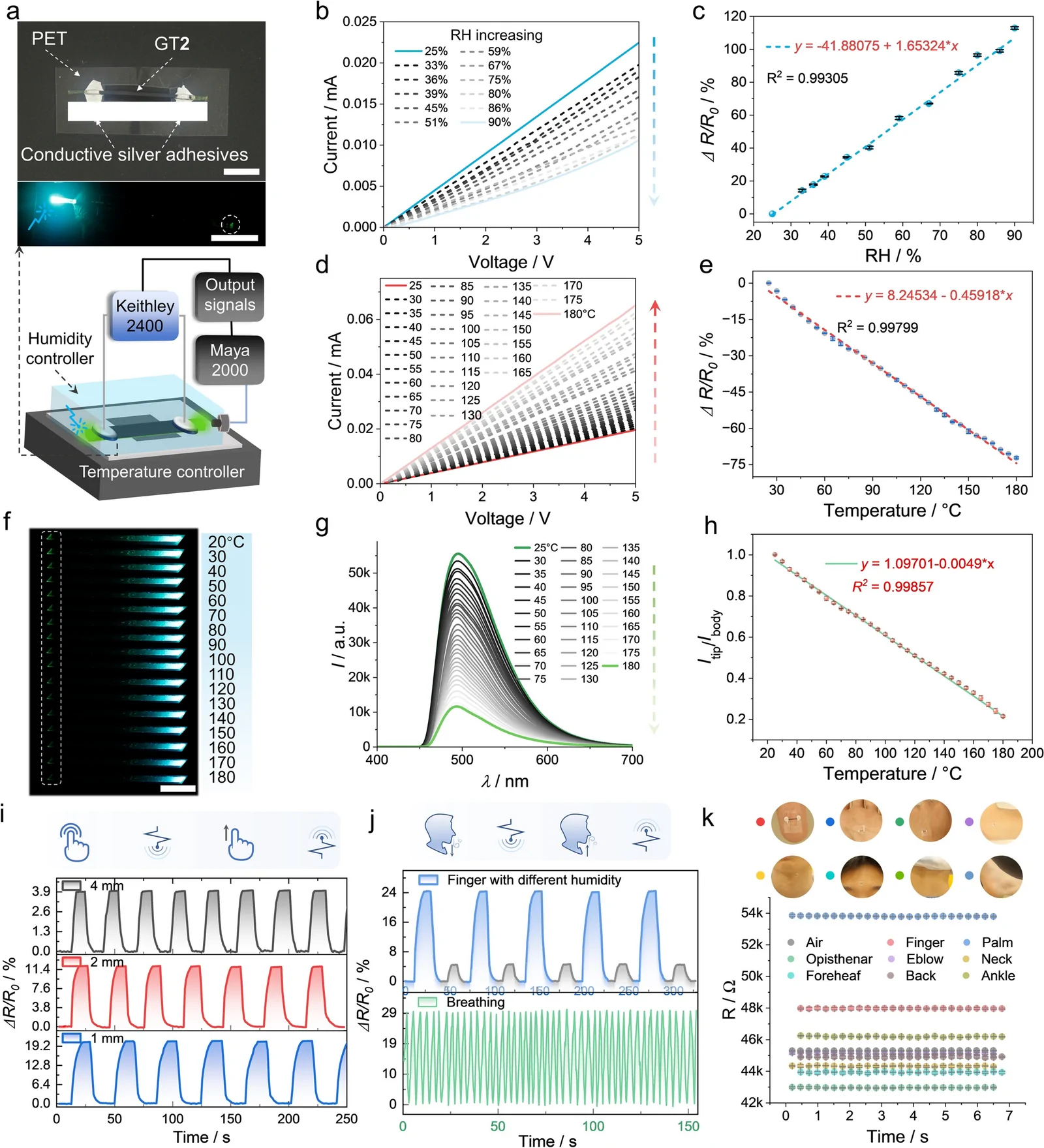
Humidity- and temperature-dependent sensing properties of hybrid crystals.** a** Schematic of the electrical signal test device integrating humidity and temperature control systems and composition of the hybrid crystal element. As one tip of the partially modified GT**2** is excited by UV light, the output signal can still be transmitted along itself to the other end.** b** *I*–*V* curves of GT**2** under a humidity range of 25 to 90% RH at 25 °C.** c** Relative resistance changes (*ΔR/R*_*0*_) as a function of temperature of GT**2** measured in three independent tests.** d** *I*–*V* curves of GT**2** measured at temperatures ranging from 25 to 180 °C under a constant relative humidity of 30% RH.** e** Linear correlation between *ΔR/R*_*0*_ and temperature obtained from three independent tests.** f** Fluorescent images of GT**2** illustrating self-waveguiding behavior at temperatures ranging from 25 to 180 °C.** g** Corresponding emission spectra collected at the crystal tip under varying temperatures and **h** linear correlation between the *I*_*tip*_/*I*_*body*_ ratio and temperature.** i** Water evaporation from a fingertip monitored by GT**2**. The fingertip repeatedly approaches the crystal at varying distances of 1 mm, 2 mm, and 4 mm.** j** Fingertip humidity signals recorded under dry air conditions and after prolonged nitrile glove wear, along with breathing signals detected by GT**2**.** k** Distinct humidity signals from different parts of the human body recorded by GT**2**. The scale bars are 5 mm in panels (**a**, **f**)

The sensing capabilities of the hybrid crystals were systematically evaluated under varying humidity and temperature conditions. As shown in Fig. 3a, a sensor element was fabricated by affixing a partially modified GT**2** crystal onto a perforated PET substrate using conductive silver adhesives. At a constant temperature of 25 °C, the resistance of GT**2** increased linearly with relative humidity (RH) ranging from 25% to 90%, exhibiting a positive humidity coefficient attributed to the multilayer structure (Figs. 3b, c and S16). This response is attributed to the multilayer structure and the hydrophilic nature of the rGO layer, which facilitates efficient moisture adsorption and desorption [53, 54]. The GT**2** sensor demonstrated excellent performance, with a coefficient of determination (*R*^2^) of 0.993, a high sensitivity of 1.65% RH^–1^, and a detection limit of 0.54% RH. When the temperature increased to 37 °C (close to human body temperature), GT**2** retained a high-humidity sensitivity of 1.59% RH^–1^, though the detection limit increased to 1.32% RH (Fig. S17). This minor degradation in performance is likely due to accelerated molecular motion at elevated temperatures, which reduces the efficiency of moisture adsorption on the sensing layer.

Conversely, when the relative humidity was held constant at 30% RH, the resistance of the GT**2** sensor decreased as the temperature increased from 25 to 180 °C (Figs. 3d and S18). This behavior is attributed to thermal excitation, which increases carrier density in the rGO layer, allowing more electrons to overcome the energy barrier, thereby reducing resistance [47, 55]. The temperature-sensing capability of GT**2** was highly linear (*R*^2^ = 0.998) with a sensitivity of 0.46% °C^–1^ and a detection limit of 1.77 °C (Fig. 3e). Under ultra-high-humidity conditions (90% RH), the temperature-sensing performance of GT**2** remained stable, albeit with a slight reduction in sensitivity (0.41% °C^–1^) and an increased detection limit (2.02 °C), likely due to excessive moisture adsorption on the rGO layer (Fig. S19). The hybrid crystals exhibit humidity- and temperature-sensing performance comparable to that of rGO/PDDA (Fig. S20). In contrast, in the absence of the rGO component, both TPU/PDDA and **2**/PDDA/TPU produce negligible electrical signals under all tested conditions. Beyond electrical sensing, fluorescence emission intensity of GT**1** and GT**2** exhibited a temperature-dependent decrease, accompanied by attenuation of self-waveguiding signals [56] (Figs. 3f, g and S21). This negative linear correlation (Fig. 3h) further validated the thermal-sensing capability of the hybrid crystals through the simultaneous measurement of optical and electrical outputs. Differential scanning calorimetry (DSC) analysis revealed that crystals **2** and GT**2** exhibit nearly identical melting (235 °C) and crystallization (216 °C) peaks, with no additional thermal transitions observed, suggesting that the hybridization does not alter the intrinsic phase behavior of the crystals (Fig. S22). Furthermore, thermogravimetric analysis (TGA) further confirms that all tested samples have initial decomposition temperatures above 180 °C (Fig. S23), demonstrating their thermal stability. These performance metrics are summarized in Table S3, which compares GT**2** with recently reported flexible sensors, highlighting its advantages in both sensitivity and operating range.

Given their excellent humidity- and temperature-sensing performance, hybrid crystal sensors hold great promise for applications in real-time monitoring of skin evaporation, respiration, and other physiological parameters. Notably, the GT**2** sensor exhibited a rapid response to water vapor evaporation, detecting changes within just 2 s when a fingertip quickly passed over it at a distance of approximately 2 mm (Fig. S24). When the fingertip was maintained at varying distances for 10 s, the sensor surface efficiently adsorbed the released vapor, demonstrating consistent response and recovery times of approximately 10 s. Moreover, the response amplitude and rate of the sensor exhibited a clear correlation with fingertip distance, effectively capturing proximity-dependent humidity variations (Fig. 3i). This capability enables the GT**2** sensor to precisely detect local fingertip humidity changes and reliably track stable respiratory patterns (Fig. 3j). Remarkably, the sensor maintains consistent responses even after prolonged exposure to high humidity, as demonstrated by stable performance following 25 days of storage at 90% RH (Fig. S25). Owing to its flexibility, high sensitivity, and portable nature, GT**2** emerges as a strong candidate for real-time breath humidity monitoring and even for distinguishing characteristic signals from different regions of the human body (Figs. 3k and S26). These capabilities pave the way for innovative applications in user authentication and low-cost physiological sensing.

### Photothermal Response and Cryogenically Controlled Motion

**Fig. 4:**
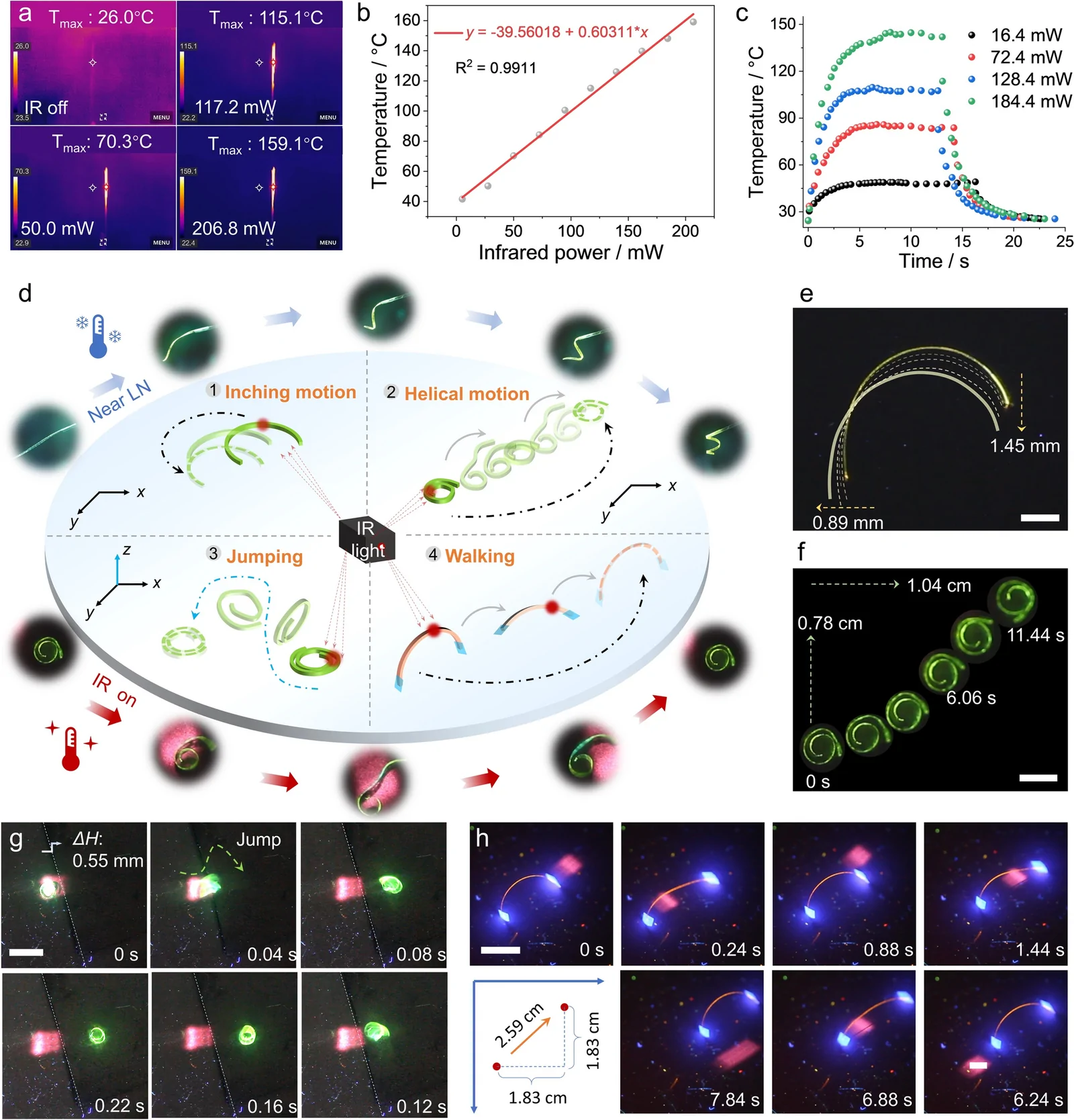
Photothermal effects and cryogenically controlled motion of hybrid crystals.** a** Infrared thermal imaging of GT**2** under different infrared (808 nm) power intensities.** b** Linear correlation between infrared power and the resulting temperature of GT**2**.** c** Heating and cooling (IR on and off) cycles of GT**2** at different infrared powers.** d** Schematic illustration of different cryogenic motion modes of hybrid crystals under infrared remote control. The top images show the sensitive curling of crystals at low temperatures. The bottom images show the cryogenic deformation of a coiled crystal under infrared irradiation. Corresponding fluorescent photographs of different cryogenic motions: **e** inching of GT**3** in a bend state; **f** helical motion of curled GT**2**; **g** jumping process of GT**2**; **h** directional walking of GT**4** equipped with paper feet. All motion processes were conducted by placing the crystal on a silicon wafer immersed in liquid nitrogen at − 150 °C. Scale bars: 5 mm in panels (**a**, **g**, **h**) and 2 mm in panels (**e**, **f**)

Beyond the sensing capabilities, we further investigated the photothermal response of the hybrid crystals to enable remote, controllable cryogenic IR light actuation. IR thermal imaging revealed that the rGO layer exhibits exceptional photothermal conversion efficiency, allowing the hybrid material to rapidly reach its threshold temperature from ambient conditions upon exposure to an 808 nm laser (Figs. 4a and S27) [57, 58]. The linear temperature–IR power relationship highlights the precise control achievable with these materials (Fig. 4b). Moreover, the heating process is both rapid and reversible, as evidenced by cooling curves following IR light source activation and deactivation (Fig. 4c). For instance, under an IR power of 128.4 mW, GT**2** reached 107 °C within 5 s and returned to room temperature within 10 s after the IR light was turned off (Movie S1). Notably, the hybrid crystal also undergoes rapid deformation in cryogenic environments—a behavior attributed to the differential thermal expansion coefficients of its constituent components [31] (Movie S2). Control experiments with individual layers confirm their distinct contributions: TPU/PDDA shows neither temperature rise nor deformation under IR irradiation; **2**/PDDA/TPU remains straight at room and high temperatures, curling only at low temperatures without additional IR effect; and rGO/PDDA exhibits significant heating under IR light but no mechanical response (Fig. S28). These results indicate that rGO governs photothermal conversion, while the crystal and TPU layers convert the thermal energy into mechanical actuation.

To quantitatively support this behavior, we characterized the thermal and structural properties of TPU and the hybrid components. DSC analysis of TPU (Fig. S29) reveals a distinct glass transition around − 40 °C, with no crystallization or melting observed up to 250 °C, confirming that TPU remains amorphous and thermally stable across the working range. The heat transfer behavior of crystal **1**, crystal **2**, and TPU was evaluated by locally irradiating a ~ 2 mm rGO-coated region at one end of each sample. Heating was highly localized in the rGO region, with adjacent areas rising by less than 10 °C, as shown in the temperature distribution profiles (Fig. S30). Additionally, length variations of crystal **1**, crystal **2**, and TPU films were measured at room temperature, − 150 °C, and 150 °C (Fig. S31). At cryogenic temperatures, TPU contracts substantially more (4.96%) than the crystals (0.89% for crystal **1** and 0.65% for crystal **2**), whereas all components show only minor expansion (0.6%–0.9%) at elevated temperatures. These results indicate that low-temperature bending arises primarily from the mismatch in thermal contraction between TPU and the crystals, while rGO-mediated photothermal heating provides localized energy to drive actuation. Consequently, we propose harnessing the sensitive photothermal response to achieve precise control over localized deformation in hybrid crystals at cryogenic conditions, thereby enabling multiple programmable motion modalities, including crawling, peristalsis, jumping, and walking (Fig. 4d). The motion of each hybrid crystal arises from the interplay of curvature, thickness, and elastic modulus [31, 32], which governs low-temperature curling under photothermal heating. Larger curling enables pronounced deformations such as meandering, crawling, or jumping, while smaller curling produces subtler inching or peristaltic motions. Surface features like end “feet” reduce friction and allow walking. The complex coupling of mechanics, thermal gradients, and friction makes quantitative modeling difficult, with low-temperature flexibility and localized IR-induced heating collectively determining the observed motion modes.

To demonstrate these motion modes, flexible crystals **3** and **4** were prepared [32, 46] and converted into hybrid crystals GT**3** and GT**4** following established protocols. When exposed to a focused IR beam at − 150 °C, these crystals exhibited controllable bending and movement driven by localized photothermal heating-induced temperature gradients (Fig. S32). The rGO layer absorbs IR radiation, locally elevating temperature and inducing expansion, which straightens the heated regions and causes shape transformation (Movie S3). Under intermittent IR radiation (206.8 mW) at − 150 °C, GT**3** exhibited repetitive bending and recovery, producing an inching motion of ~ 1 mm over 26 s; while the 720° curled GT**2** achieved meandering crawling of ~ 1 cm within 11 s (Fig. 4e, f; Movie S4). As expected, concentrated photothermal energy (408.4 mW) induced rapid expansion and contraction, allowing GT**2** to execute jumping motions on the silicon-based surface (height: 0.55 mm; Movie S5 and Fig. S33). Notably, it could leap over obstacles in just 0.22 s, tracing a parabolic trajectory with a height of ~ 2 mm and a length of ~ 8 mm (Fig. 4g). These motions stem from the photothermal effect, inducing a temperature gradient within the crystal structure, leading to asymmetrical deformation, with greater coiling at lower temperatures corresponding to more intense photothermal motion. Additionally, GT**4**, with directional control via remote IR light (206.8 mW), exhibited a walking motion, further showcasing the versatility of these materials in dynamic applications (Fig. 4h; Movie S6). The intrinsic flexibility of the crystals at low temperatures [31, 56] allows for significant deformation without structural damage, ensuring sustained movement. These results demonstrate the tremendous potential of hybrid crystals for remote infrared-controlled actuation, particularly in cryogenic conditions where traditional materials often fail [59, 60], with Table S4 offering a comparison to representative flexible actuators.

### Cryogenic Signal Modulation and Stability

**Fig. 5:**
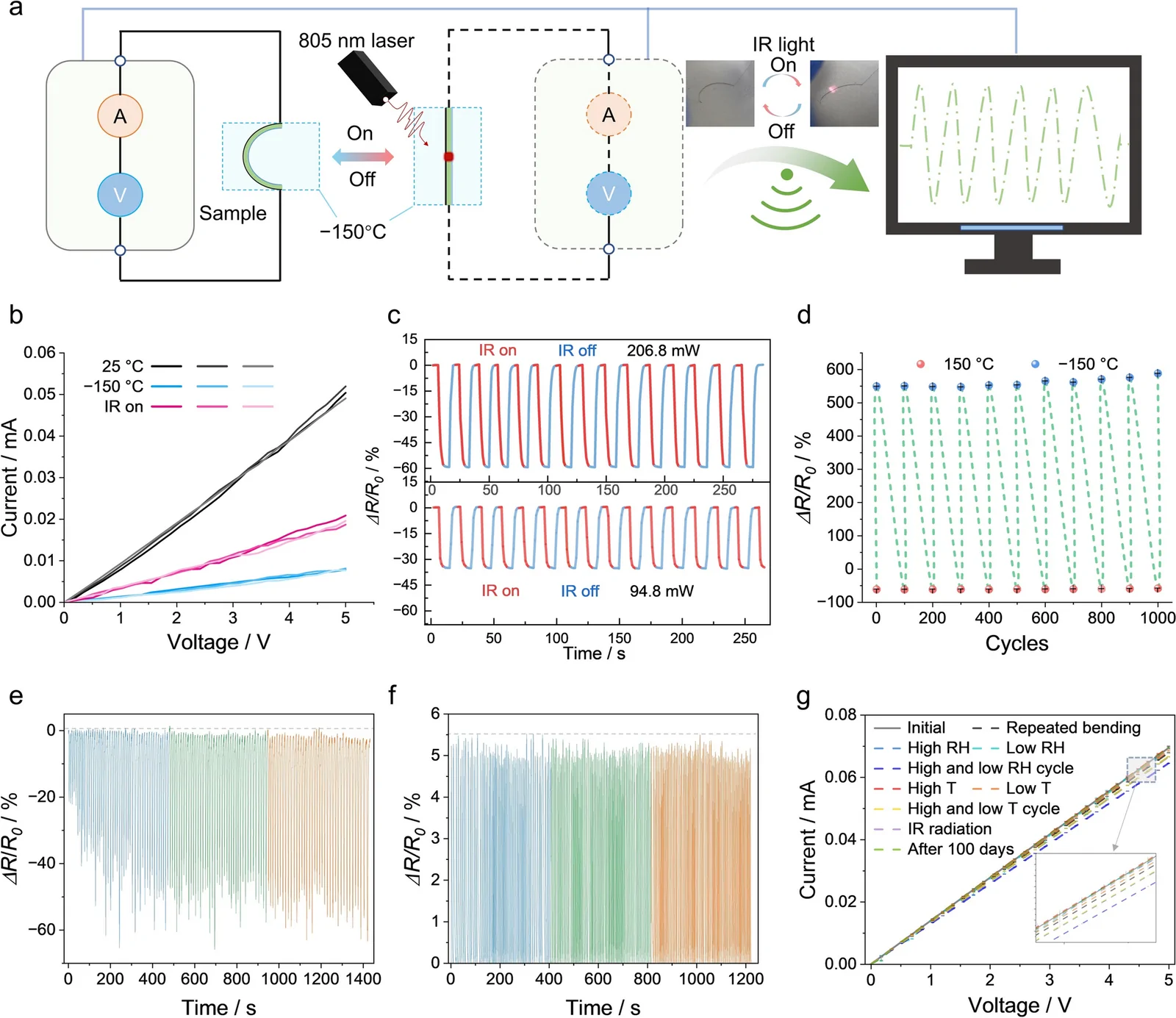
Signal output of photothermal excitation and fatigue properties of hybrid crystals.** a** Schematic of output signals of the hybrid crystal repeatedly irradiated by infrared light in cryogenic environments. The inset shows GT**2** bending at − 150 °C, with deformation decreasing under infrared irradiation and recovering after the irradiation is turned off.** b** *I*–*V* curves of GT**2** at 25 °C, − 150 °C, and − 150 °C with infrared radiation (206.8 mW).** c** Reversible response signals of GT**2** measured over time with periodic infrared (IR) irradiation at power intensities of 206.8 mW (top) and 94.8 mW (bottom).** d** Fatigue cycles of GT**2** under high- and low-temperature switching.** e** 126 fatigue cycles of repeated infrared irradiation (206.8 mW) on GT**2** at 25 °C and 10% RH conditions. The variation in peak values is caused by different infrared irradiation times, leading to varying temperatures reached by the sample.** f** 200 fatigue cycles of GT**2** under small humidity variations (achieved by quick finger proximity).** g** *I*–*V* curves of the same GT**2** in different environments, indicating its stability

Building upon the demonstrated photothermal response and sensing capabilities, we further integrated cryogenic actuation and thermal sensing into a photothermally driven signal output system based on the hybrid crystal (Fig. 5a). Electrical evaluations at room temperature, − 150 °C, and − 150 °C with IR radiation conditions confirmed the stable electrical properties across varying thermal and IR exposure conditions (Fig. 5b). Under repeated IR irradiation in a cryogenic environment, GT**2** exhibited a stable and reversible resistance response, with resistance decreasing upon IR exposure and recovering upon its removal (Fig. 5c). Notably, the response intensity exhibited a clear dependence on the irradiation strength, highlighting the tunable nature of its signal modulation under varying IR conditions. This performance reflects the high photothermal conversion efficiency and robustness of GT**2** in extreme environments, further supporting its applicability in photothermally controlled actuation systems. Fatigue tests under alternating high- and low-temperature conditions confirmed its resilience, with stable performance regardless of the thermal cycling regime or localized IR exposure, indicating persistent photothermal-driven behavior (Figs. 5d, e and S34). Although minor variations in peak temperature were observed due to differences in IR exposure time, the overall stability of the material remained unaffected. In addition, under small-scale humidity variations, such as rapid finger proximity, the crystal maintained stable performance over 200 fatigue cycles, showcasing its ability to operate reliably under dynamic environmental conditions (Fig. 5f). Even after undergoing mechanical bending, humidity changes, temperature cycling, repeated IR irradiation, and prolonged storage, GT**2** retained consistent electrical signal output, with deviations not exceeding 8% from its original state (Figs. 5g and S35). The structural stability of the multilayer system was further evaluated under cycles of high/low humidity (20%–90% RH), high/low temperature (– 150 to 150 °C), and repeated infrared irradiation. XPS spectra of **2**/PDDA, rGO/PDDA, and TPU/PDDA layers exhibited binding energy shifts of less than 0.05 eV, confirming that the interfacial chemical states and electronic environments remained largely unchanged (Fig. S2). The slight peak shape and intensity variations are more reasonably attributed to reversible surface adsorption/desorption or local chemical environment adjustments, rather than new bond formation or chemical degradation. AFM images of the crystal surface and PDDA-coated layer confirm that surface morphology is preserved after cycling (Fig. S6). Moreover, AFM analysis of the TPU and rGO layers, together with SEM images of GT**1** and GT**2** before and after cycling, reveals that the sandwich-like architecture remains intact without microfracture or delamination (Figs. S5 and S36). These observations highlight the excellent interfacial and structural integrity of the multilayer hybrid crystals under repeated extreme condition. The seamless integration of mechanical durability, environmental responsiveness, and stable electrical performance underscores the potential of hybrid crystals for advanced applications in soft actuators, environmental sensors, and multifunctional adaptive devices [61]. With further optimization, these materials hold promise for addressing challenges in complex application scenarios, paving the way for next-generation flexible and responsive systems.

## Conclusions

In this study, we successfully developed multifunctional hybrid crystals that seamlessly integrate flexible sensing and cryogenic photothermal responsiveness into a unified platform. By strategically combining a flexible organic crystal with rGO and TPU, we engineered a layered architecture in which each component plays a distinct yet complementary role. The hybrid material leverages the fluorescence and electrical conductivity provided by organic crystal and rGO, to realize dual humidity–temperature sensing, while the strong photothermal response of rGO, combined with the intrinsic low-temperature adaptability of the TPU-integrated crystal, enables stable and controlled cryogenic actuation. Importantly, fluorescence sensing from the crystalline layer and electrical sensing from the rGO network operate in a cooperative rather than competitive manner, with photothermal effects bridging the two pathways. While this work represents notable progress toward multifunctional integration, it may not fully achieve comprehensive optimization across all functional domains. Looking ahead, establishing quantitative correlations between fluorescence and conductivity under combined stimuli will provide a foundation for further advancing hybrid crystalline systems as versatile platforms for multimodal sensing in adaptive technologies.

## Supplementary Information

Below is the link to the electronic supplementary material.

Supplementary file1 (MP4 682 KB)

Supplementary file2 (MP4 5392 KB)

Supplementary file3 (MP4 752 KB)

Supplementary file4 (MP4 3468 KB)

Supplementary file5 (MP4 1137 KB)

Supplementary file6 (MP4 1361 KB)

Supplementary file7 (DOCX 14131 KB)
