# Supplementary material for: Sandwich-Architected Hybrid Organic Crystals with Humidity–Temperature Sensing and Cryogenic Photothermal Actuation
Source: Nanomicro Lett. 2026 Jan 5;18:160. doi: 10.1007/s40820-025-01996-7 (PMC12765820; doi:10.1007/s40820-025-01996-7)
Supplement: Supplementary file 7 — Supplementary file7 (DOCX 14131 KB) [file 40820_2025_1996_MOESM7_ESM.docx]

Supporting Information for

**Sandwich-Architected Hybrid Organic Crystals with Humidity-Temperature Sensing and Cryogenic Photothermal Actuation**

Linfeng Lan^1,2^, Lijie Wang^1*^, Chenguang Wang^1^, Hongyu Zhang^2*^

^1^ State Key Laboratory of Integrated Optoelectronics, College of Electronic Science and Engineering, Jilin University, Qianjin Street, Changchun 130012, P. R. China

^2^ State Key Laboratory of Supramolecular Structure and Materials, College of Chemistry, Jilin University, Qianjin Street, Changchun 130012, P. R. China

*Corresponding authors. E-mail: [wang_lij@jlu.edu.cn](mailto:wang_lij@jlu.edu.cn) (Lijie Wang); [hongyuzhang@jlu.edu.cn](mailto:hongyuzhang@jlu.edu.cn) (Hongyu Zhang)

**S1 Supplementary Scheme and Figures**

**Scheme S1** Molecular formulas for compounds **1**–**4**


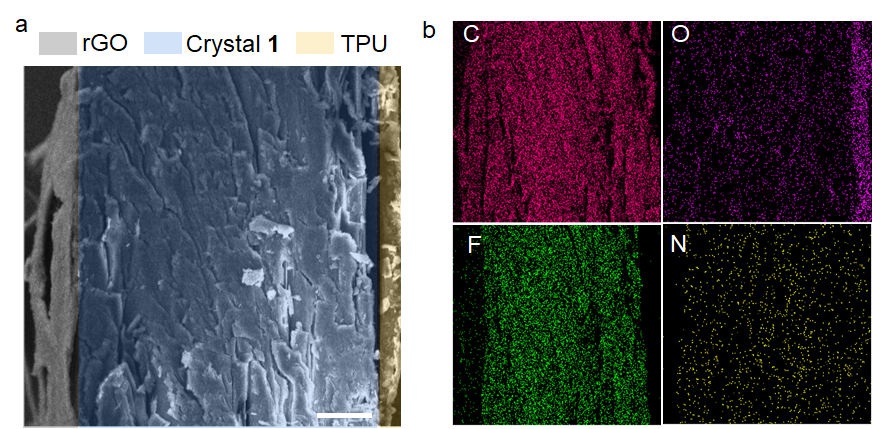


**Fig. S1** Scanning electron microscopy (SEM) images (**a**) and Energy-dispersive X-ray spectroscopy (EDX) elemental maps (**b**) of the GT**1** showing the spatial distribution of the constituent elements. The scale bar is 20 μm


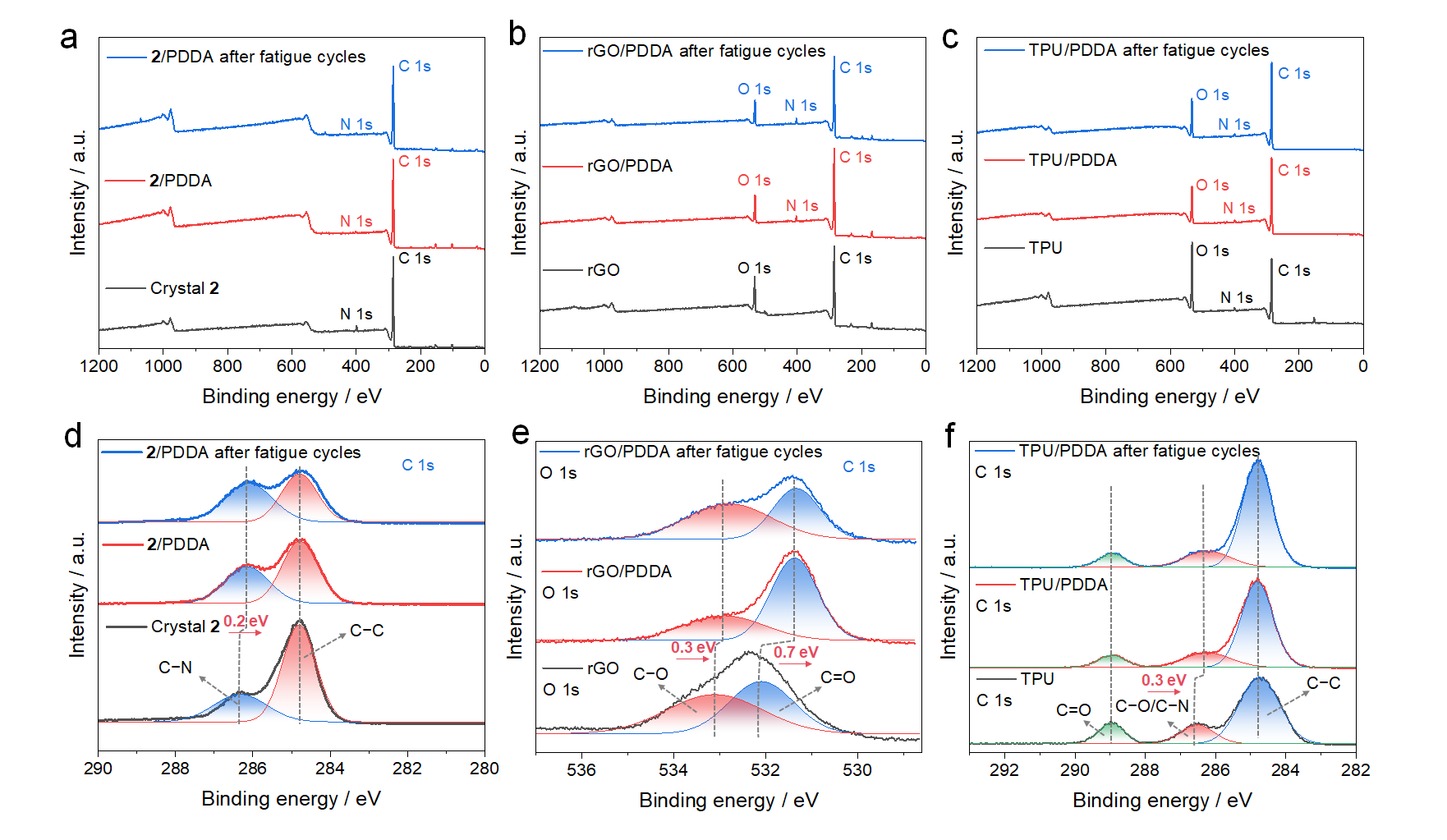


**Fig. S2** (**a, d**) Wide-scan and C 1s XPS spectra of crystal **2**, **2**/PDDA, and **2**/PDDA after the fatigue cycles. (**b, e**) Wide-scan and O 1s XPS spectra of rGO, rGO/PDDA, and rGO/PDDA after the fatigue cycles. (**c, f**) Wide-scan and C 1s XPS spectra of TPU, TPU/PDDA, and TPU/PDDA after the fatigue cycle. The fatigue cycles refer to repeated operation of the samples under alternating conditions of high/low humidity (20–90% RH), high/low temperature (–150 to 150°C), and repeated infrared irradiation


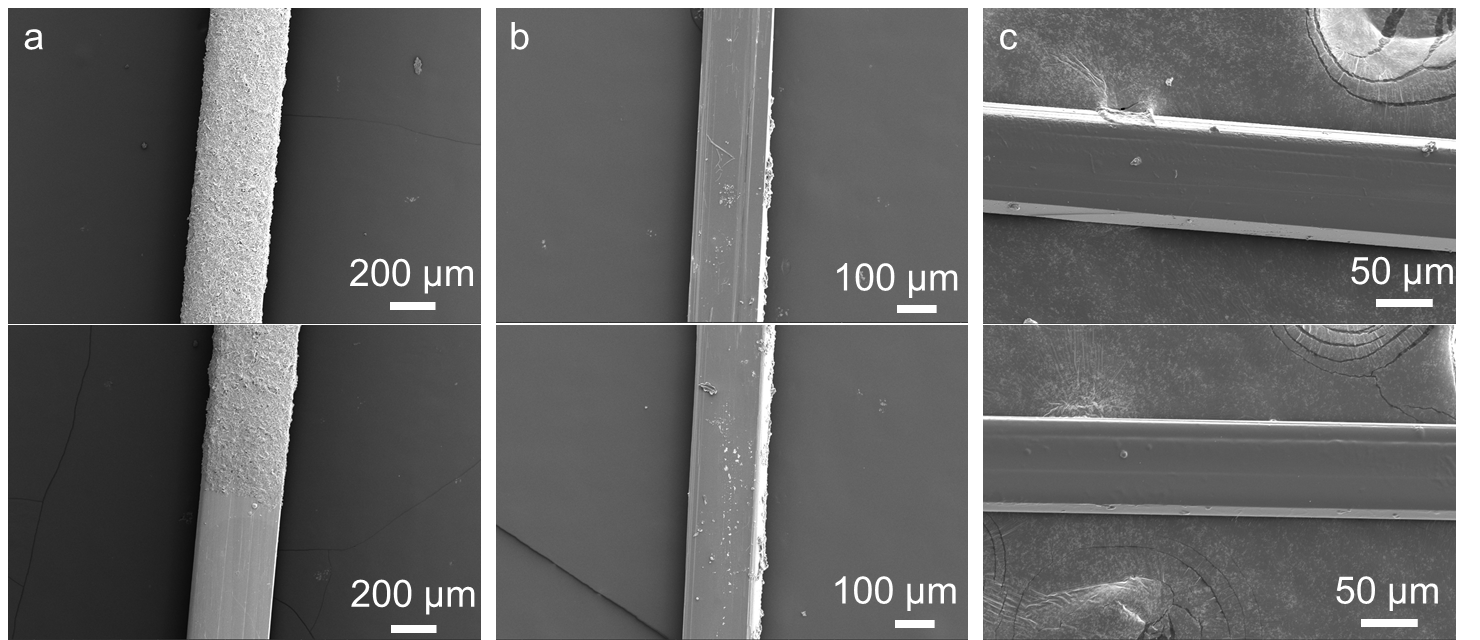


**Fig. S3** SEM images of hybrid crystals GT**1** and GT**2**: (**a**) Surface morphology of the rGO-modified side and junction region, showing uniform coating and roughness distribution. (**b**) Backside of the rGO-modified crystal, showing its original morphology without surface coating. (**c**) Surface morphology of the TPU-modified side, illustrating the smooth and continuous coating


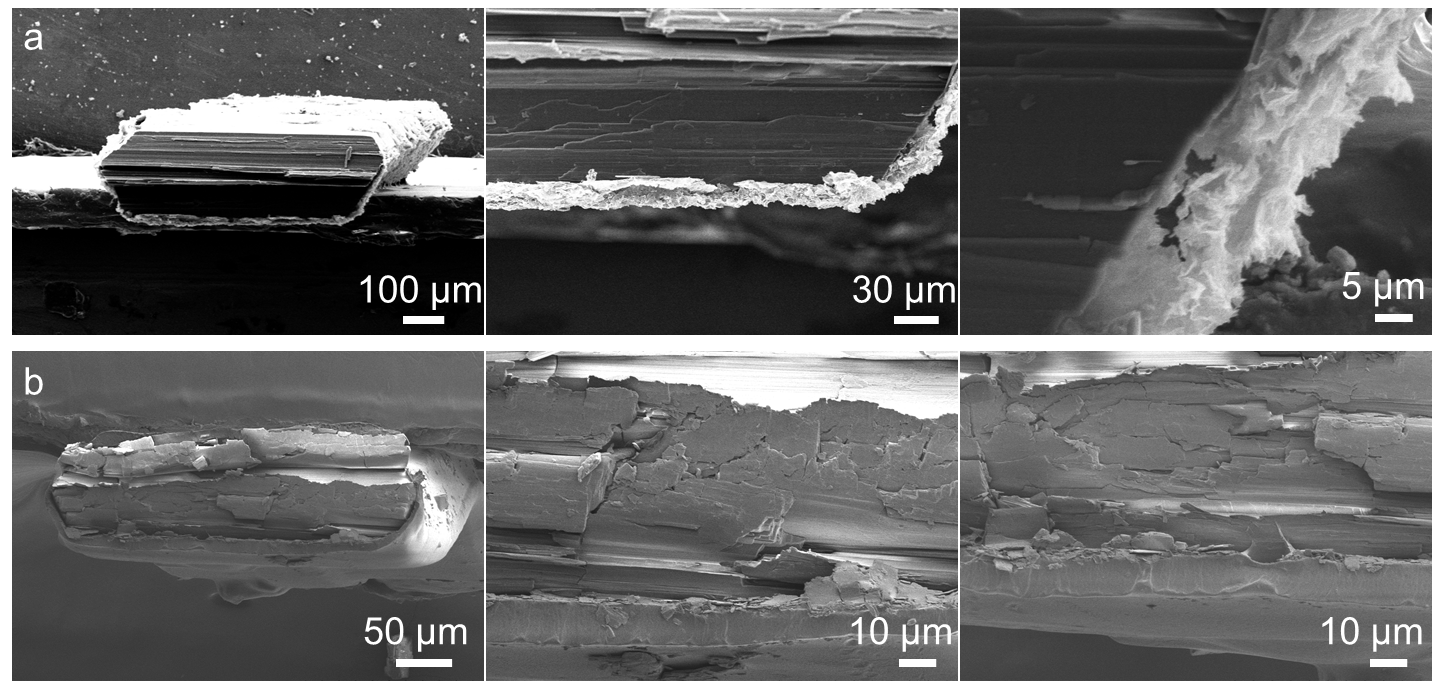


**Fig. S4** High-magnification SEM images of the hybrid crystal cross-sections: (**a**) Morphology and thickness of the rGO layer, showing its uniform coating and layered structure. (**b**) Morphology and thickness of the TPU layer, illustrating the smooth surface and compact adhesion to the crystal substrate


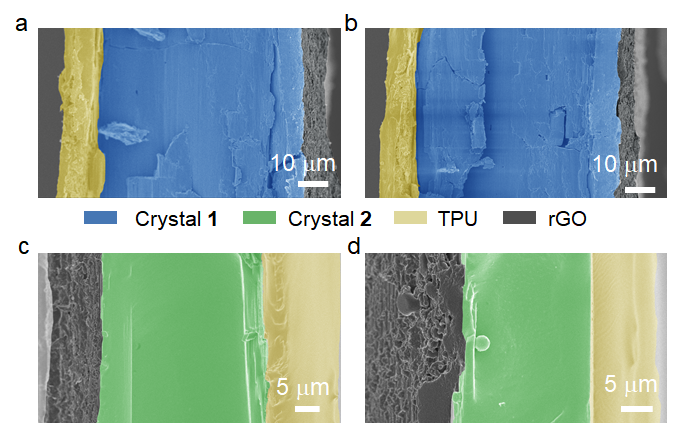


**Fig. S5** Cross-sectional SEM images of the hybrid crystals: (**a, c**) GT**1** (**a**) and GT**2** (**c**), showing the distinct sandwich-like multilayer structure; (**b, d**) GT**1** (**b**) and GT**2** (**d**) after cycles of high/low humidity (20% to 90% RH), high/low temperature (–150 to 150 °C), and repeated infrared irradiation, demonstrating the structural stability of the multilayer structure


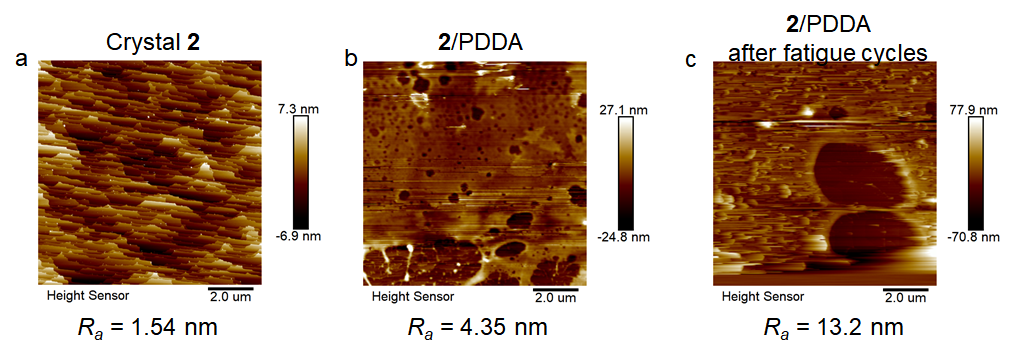


**Fig. S6** (**a, b**) AFM images of the surface morphology of crystal **2** and **2**/PDDA, showing the crystal surface and the deposited PDDA layer. (**c**) AFM image of **2**/PDDA after cycles of high/low humidity (20% to 90% RH), high/low temperature (–150 to 150°C), and repeated infrared irradiation


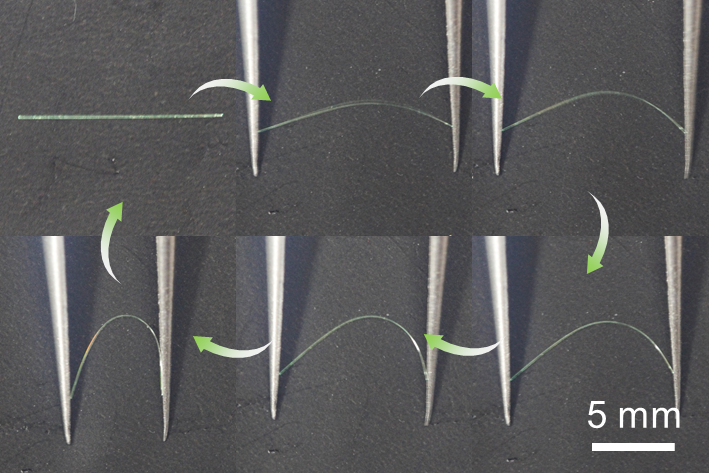


**Fig. S7** Reversible elastic bending process of the pristine crystal **2**


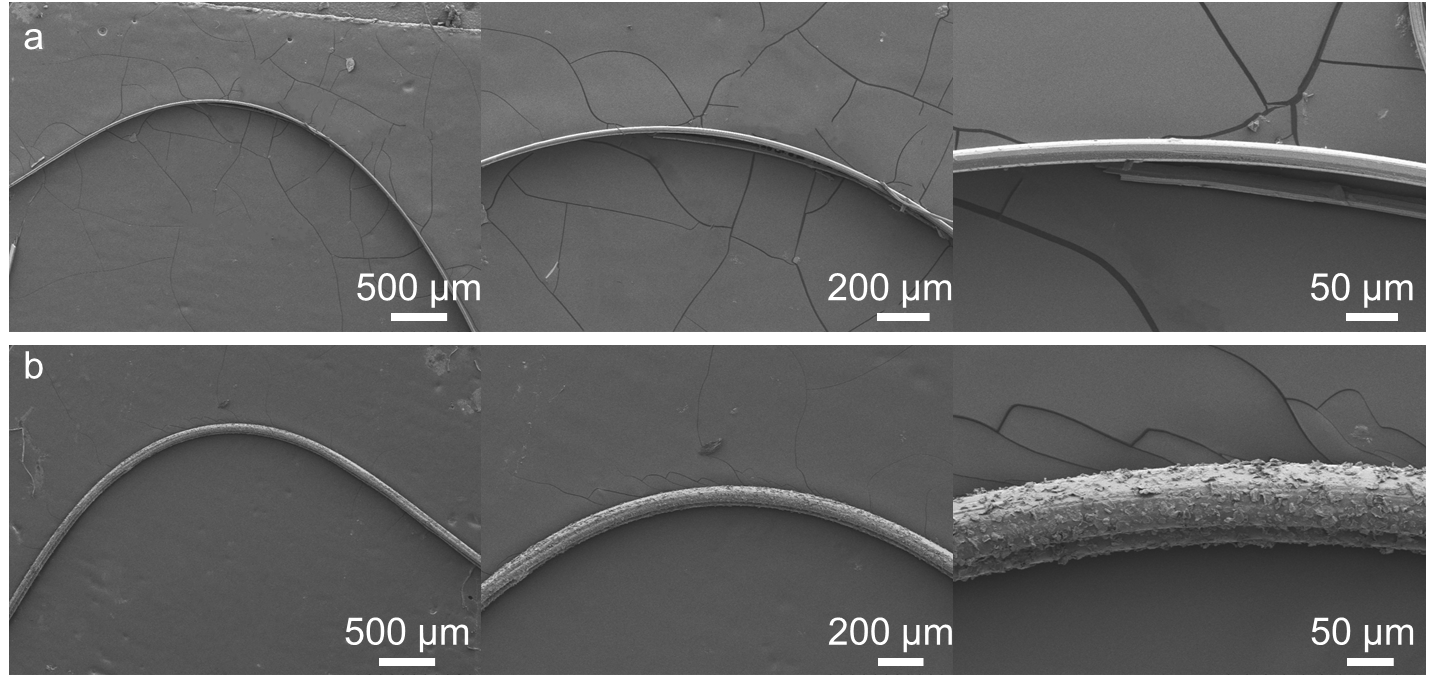


**Fig. S8** (**a, b**) SEM images of the pristine crystal **2** (**a**) and hybrid crystal GT**2** (**b**) under bending deformation showing intact structure under external forces


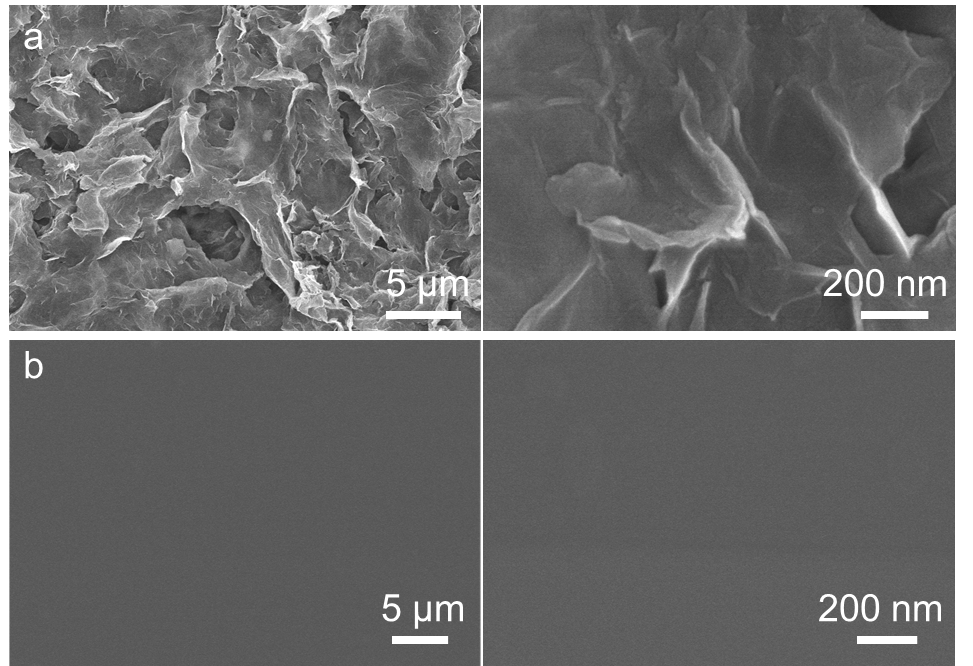


**Fig. S9** High-magnification field-emission scanning electron microscopy (FE-SEM) images of hybrid crystal GT**2** surfaces after 1000 cycles of repeated bending: (**a**) Surface morphology of the rGO layer, showing visible wrinkles and structural integrity. (**b**) Surface morphology of the TPU layer, exhibiting smooth and undamaged surface characteristics


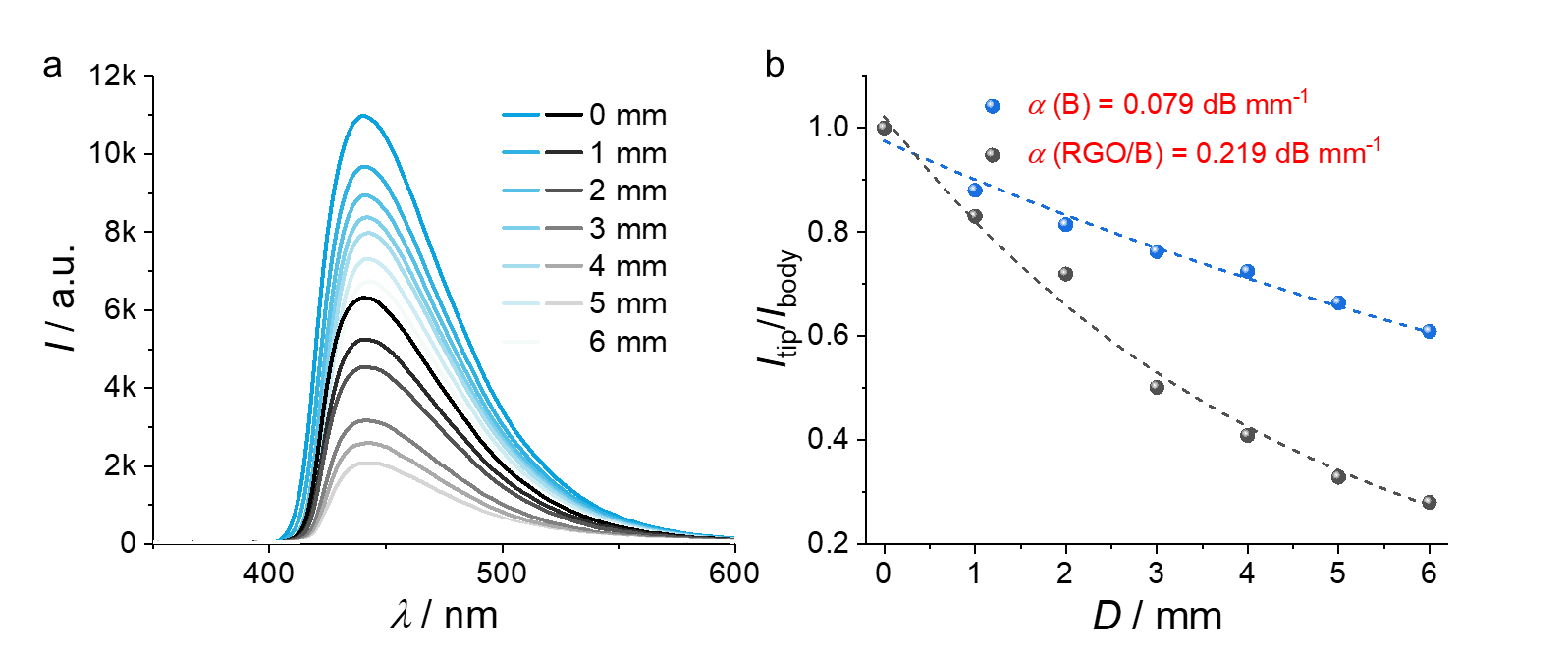


**Fig. S10** The optical waveguides properties of a crystal GT**1**: (**a**) Fluorescence spectra collected at one tip of GT**1** with different distances between the tip and the excitation site of the laser. (**b**) The *I*_tip_/*I*_body_ decays of GT**1**. The optical loss coefficients (α) are determined by a single-exponential fitting of the function. *I*_tip_/*I*_body_ = *A*exp(–α*D*), in which *I*_tip_ and *I*_body_ are the fluorescence intensities of out-coupled and incidence light respectively, *A* is the optical loss coefficient and *D* is the distance between the excited site and the tip of crystals for collecting emission


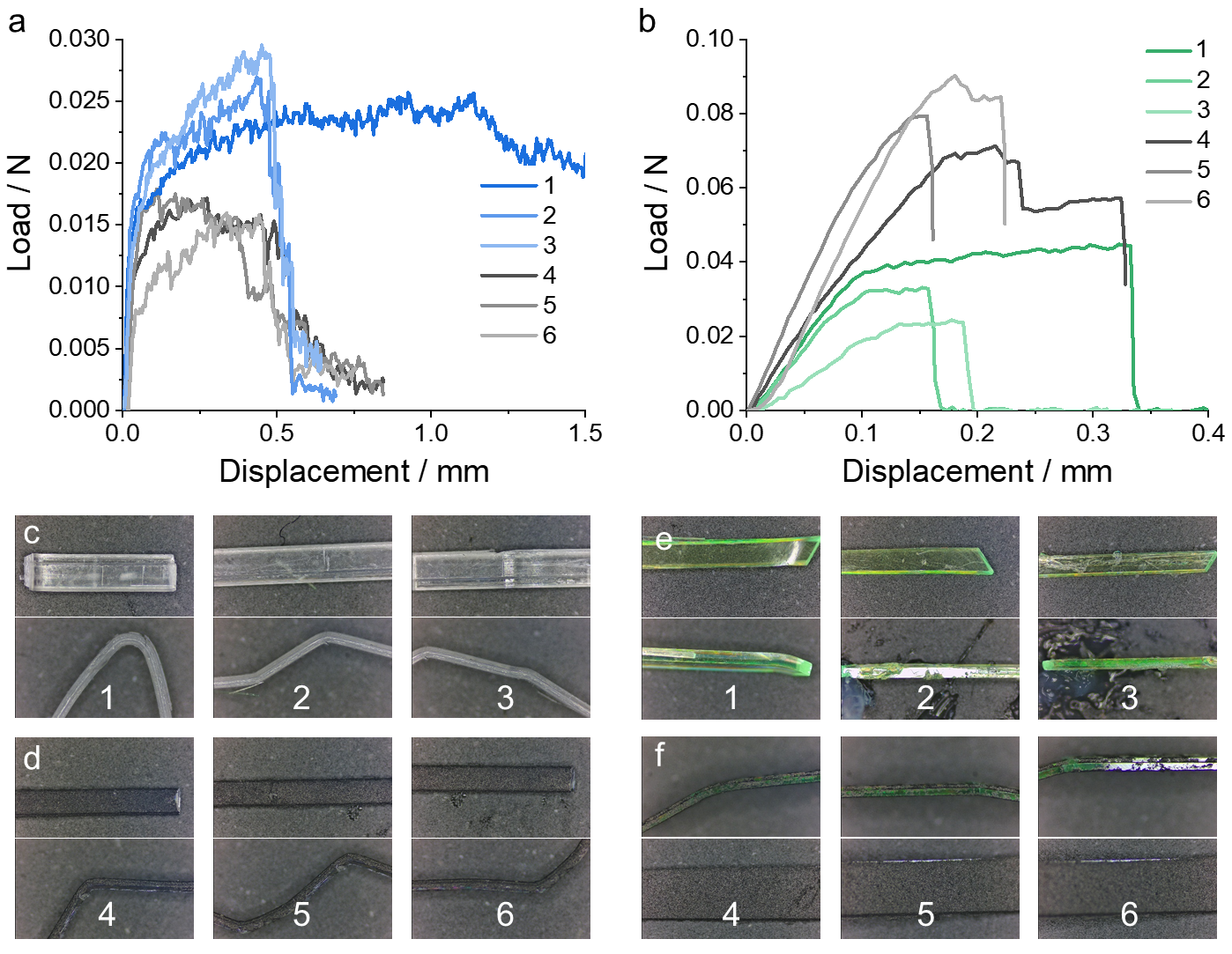


**Fig. S11** (**a, b**) Load–displacement curves obtained from the three-point bending test of crystals **1** and GT**1** (**a**), **2** and GT**2** (**b**). (**c–f**) Optical microscopy images for measuring the width and thickness of samples **1** (**c**), GT**1** (**d**), **2** (**e**), and GT**2** (**f**). The corresponding values of width and thickness are summarized in Table S1.


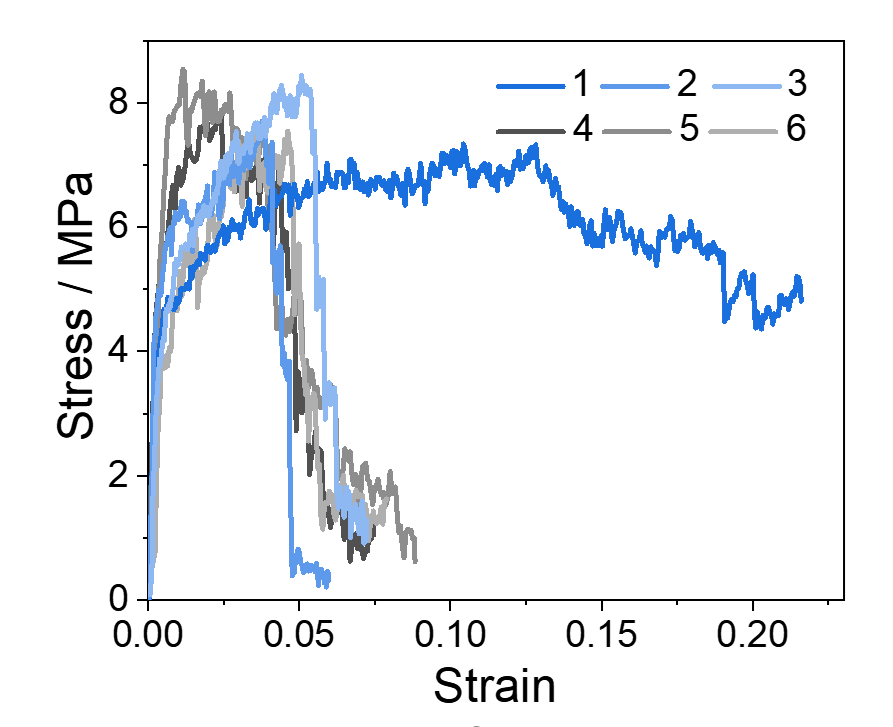


**Fig. S12** Stress–strain curves obtained from the three-point bending tests of crystals **1** (Samples 1–3) and GT**1** (Samples 4–6)


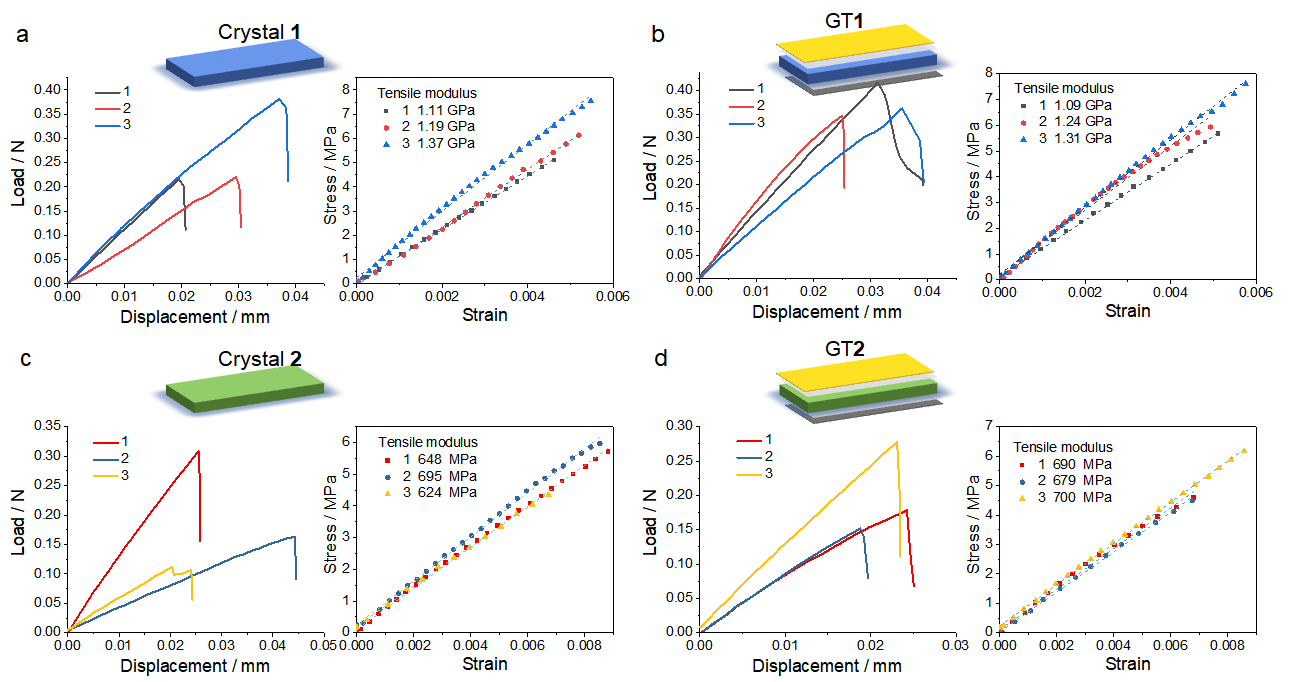


**Fig. S13** Displacement–load curves and corresponding stress–strain curves of pristine crystal **1** (**a**), GT**1** (**b**), pristine crystal **2** (**c**), and GT**2** (**d**) under tensile testing. Three independent specimens were tested for each sample


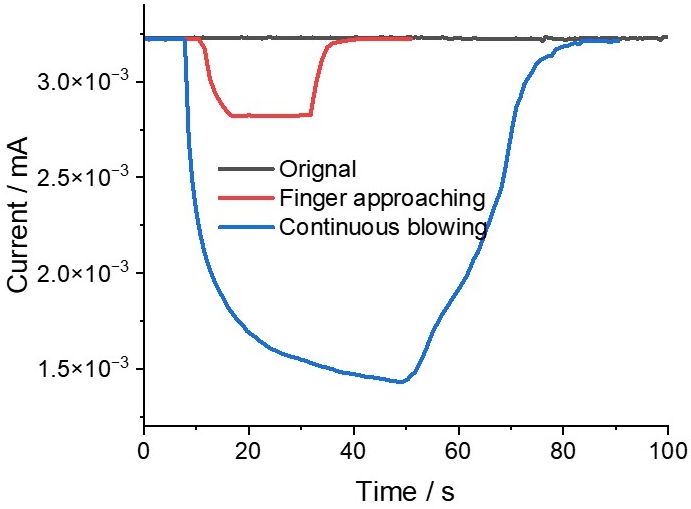


**Fig. S14** Current variation over time for GT**2** under initial conditions, finger proximity, and blowing conditions, corresponding to Fig. 2k


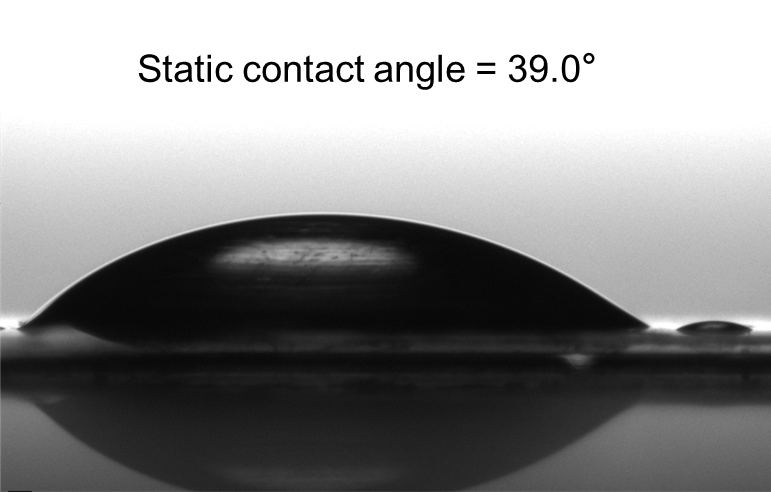


**Fig. S15** Surface tension of water drops on the rGO layer of GT**2**


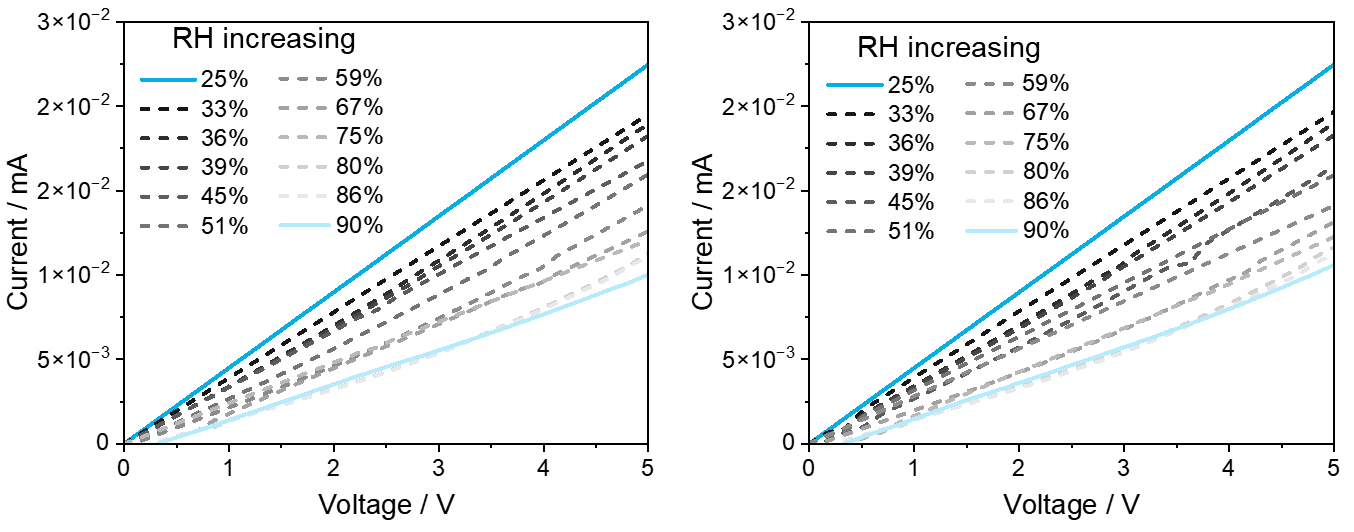


**Fig. S16** The second (left) and third (right) tests demonstrate the electrical response of GT**2** to varying relative humidity (RH) levels at 25°C


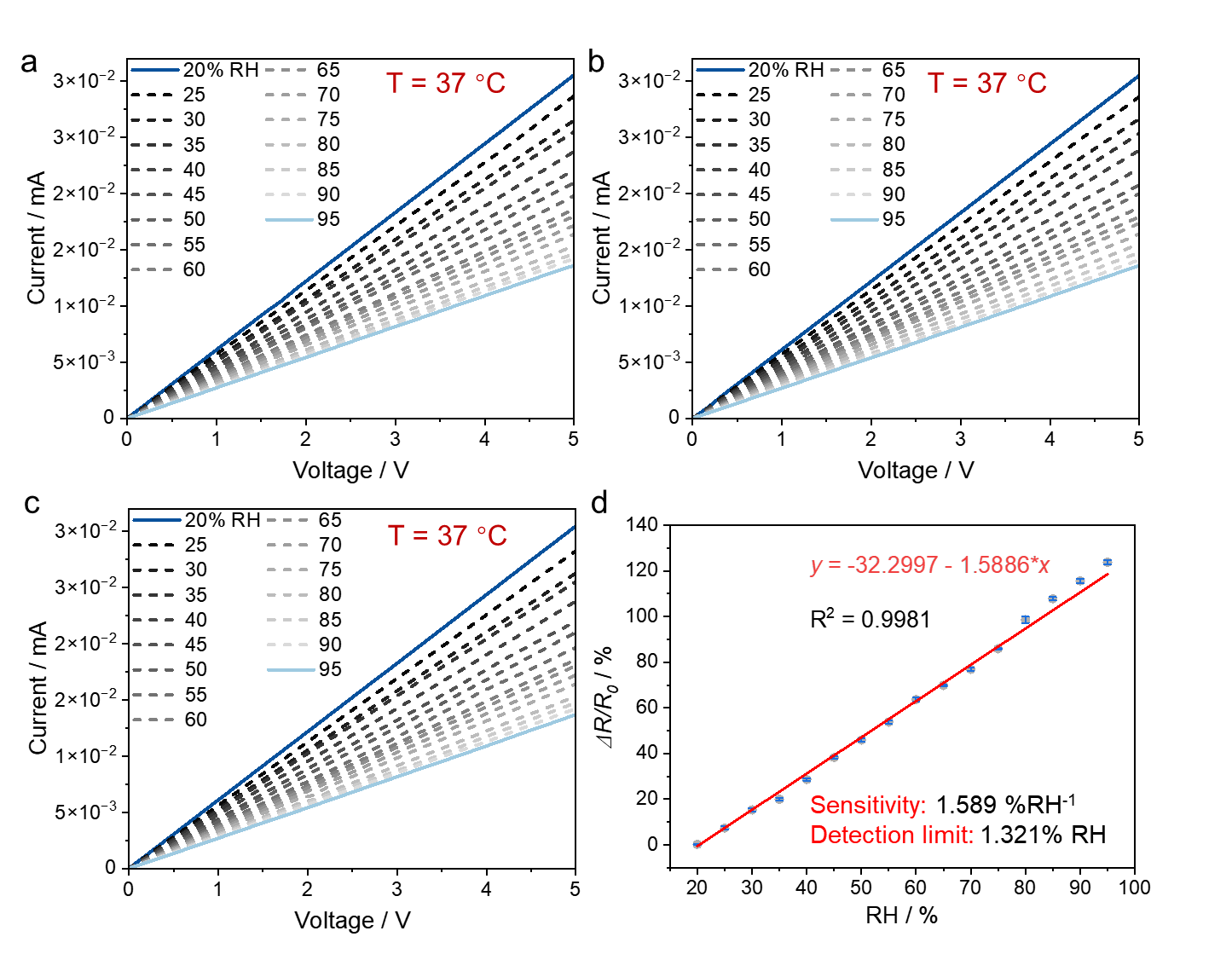


**Fig. S17** (**a–c**) The current-voltage (*I*-*V*) curves of GT**2** under different relative humidity (RH) conditions at 37°C, measured repeatedly over three cycles to confirm reproducibility. (**d**) The corresponding linear correlation between normalized resistance change (*ΔR/R_0_*) and RH


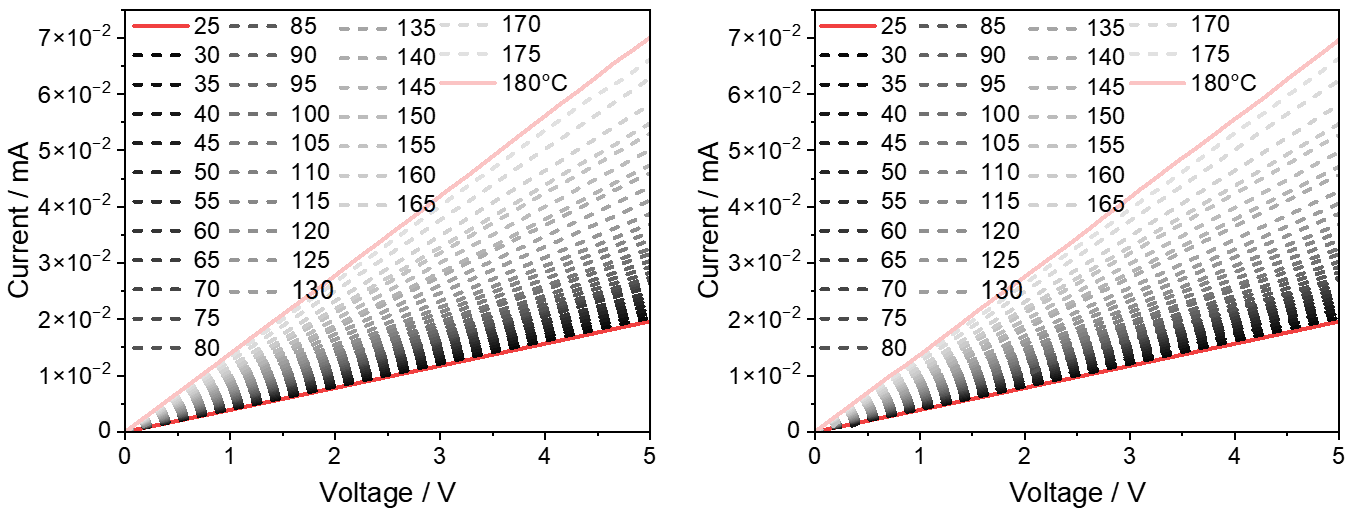


**Fig. S18** The second (left) and third (right) tests demonstrate the electrical response of GT**2** to varying temperature levels at 30% RH


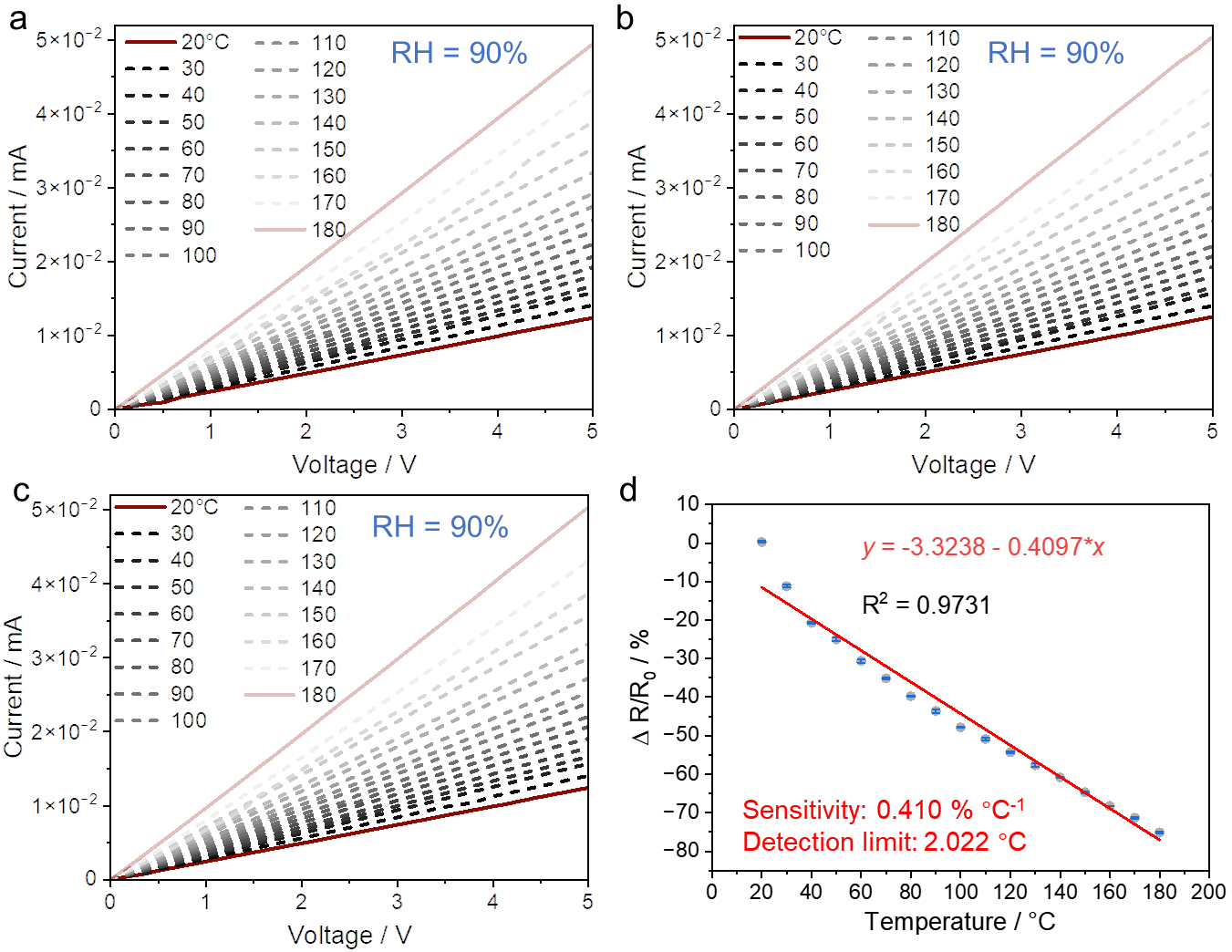


**Fig. S19** (**a–c**) The *I*-*V* curves of GT**2** under different temperature conditions at 90% RH, measured repeatedly over three cycles to confirm reproducibility. (**d**) The corresponding linear correlation between normalized resistance change (*ΔR/R_0_*) and temperature


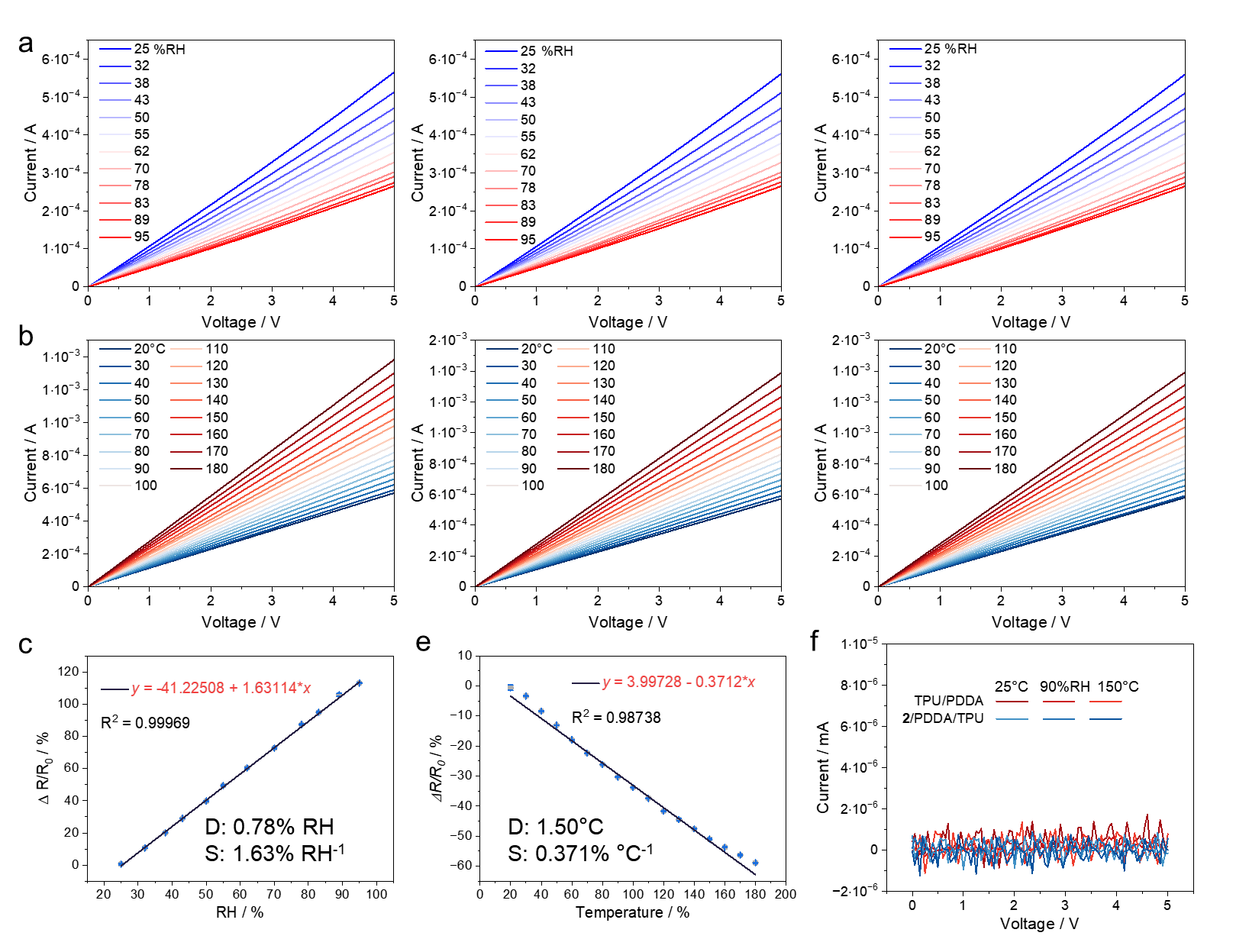


**Fig. S20** (**a**) The *I*-*V* curves of rGO under different relative humidity conditions at 25°C, measured repeatedly over three cycles. (**b**) The *I*-*V* curves of rGO under different temperature conditions at 30% RH, measured repeatedly over three cycles. (**c**) The corresponding linear correlation between normalized resistance change (*ΔR/R_0_*) and relative humidity. (**e**) The corresponding linear correlation between normalized resistance change (*ΔR/R_0_*) and temperature. (**f**) The *I*-*V* curves of TPU/PDDA and **2**/PDDA/TPU under different conditions


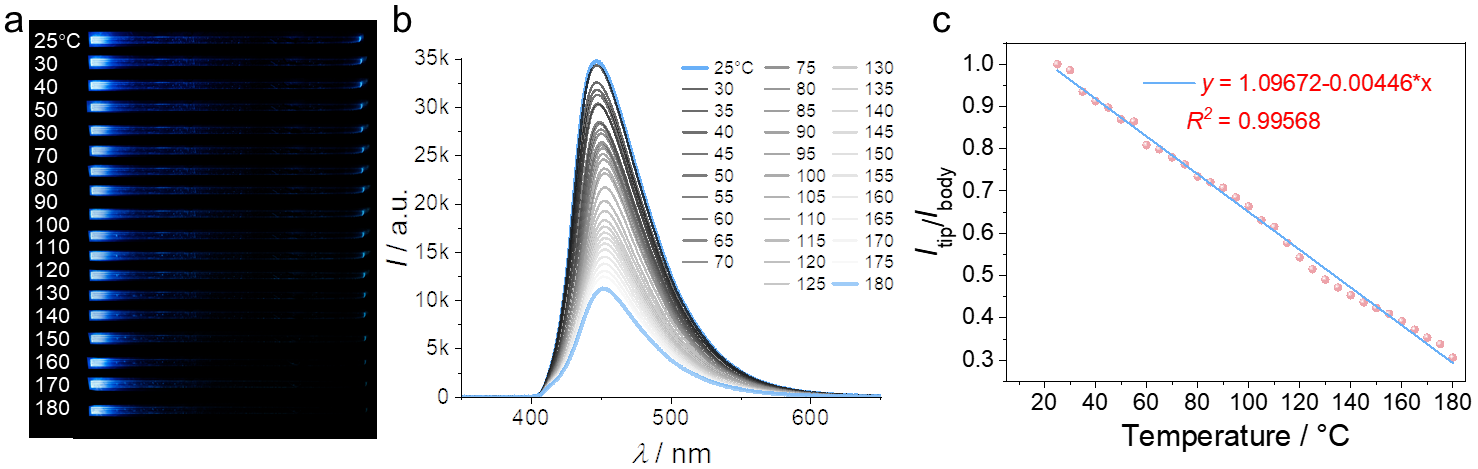


**Fig. S21** (**a**) Fluorescent images of GT**1** showing optical waveguides at temperatures ranging from 25°C to 180°C. (**b**) Emission spectra collected at the crystal tip under varying temperatures. (**c**) Linear correlation between the ratio of *I_tip_*/*I_body_* and temperature, demonstrating the temperature-dependent attenuation of optical signals


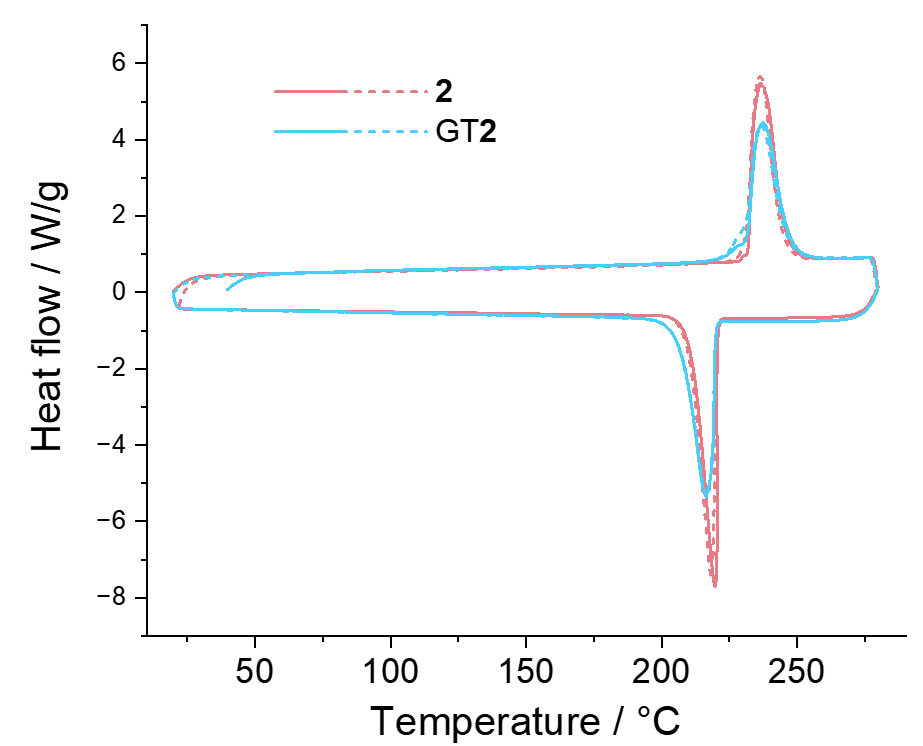


**Fig. S22** Differential scanning calorimetry (DSC) curves of crystals **2** and GT**2**. The DSC curves


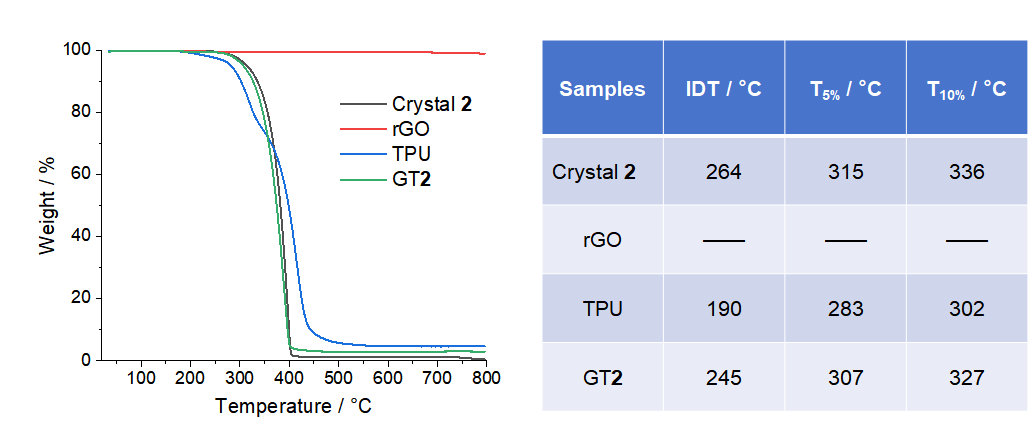


**Fig. S23** TGA curves of crystal **2**, rGO, TPU, and GT**2**, and summary table of thermal parameters, including Initial decomposition temperature (IDT), decomposition temperature at 5% weight loss (T), and decomposition temperature at 10% weight loss (T_10%_)


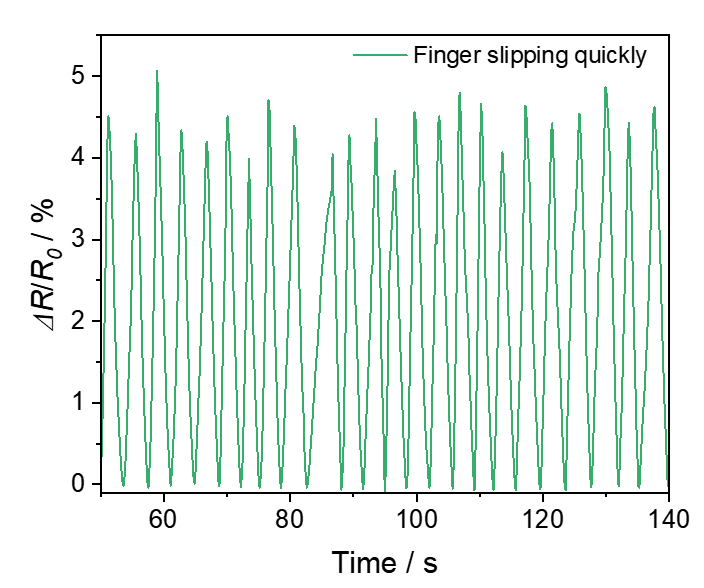


**Fig. S24** Monitoring water evaporation from finger recorded by GT**2**. A Finger quickly and repeatedly passing over GT2 at approximately 2 mm height


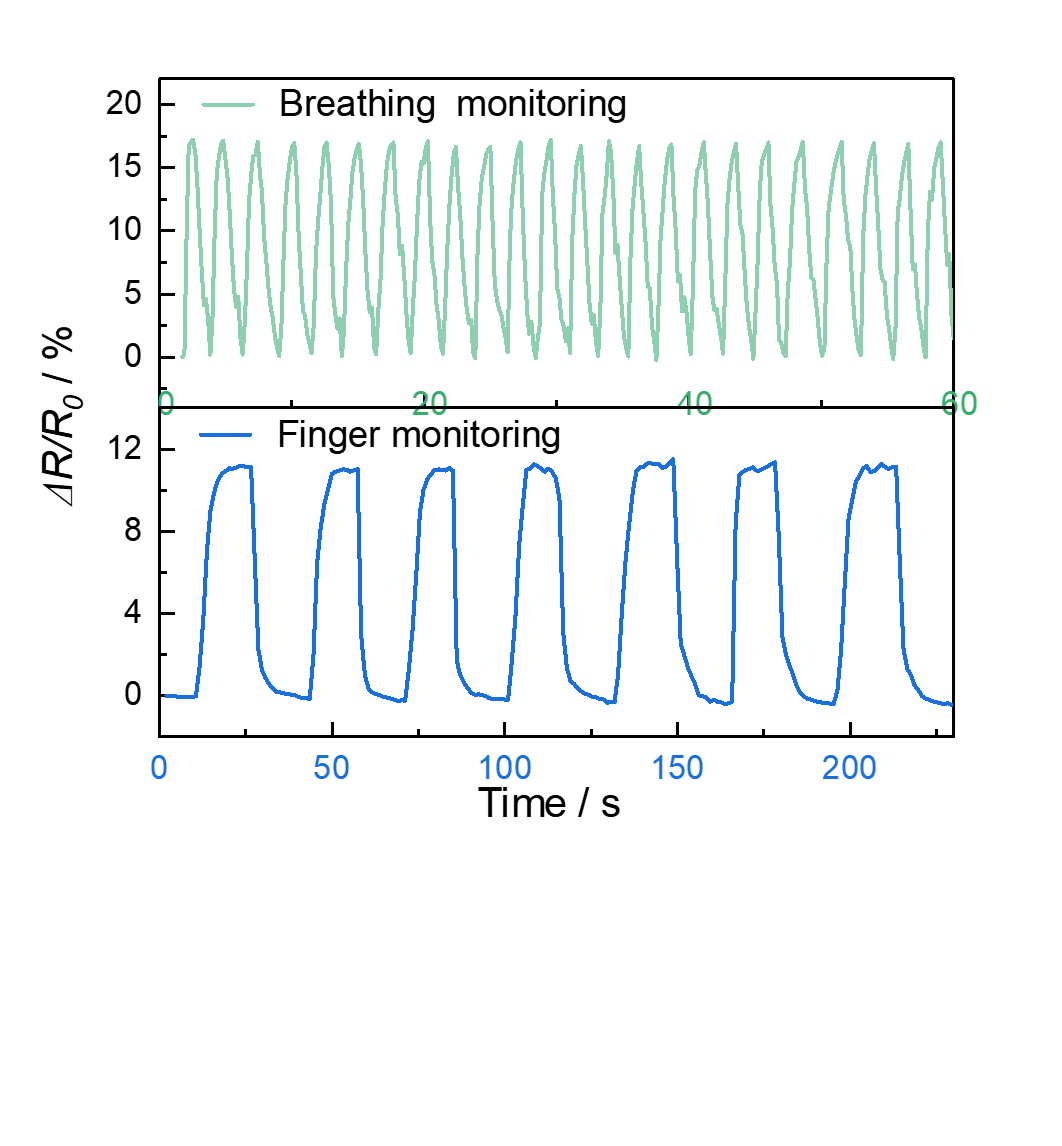


**Fig. S25** Humidity sensing performance of GT**2** after 25 days of storage at 90% RH followed by drying at 60°C for 2 h. Top: detected breathing signals. Bottom: local fingertip moisture changes monitored by GT**2**, with the fingertip repeatedly approaching and moving away from the sensor (~4 mm distance)


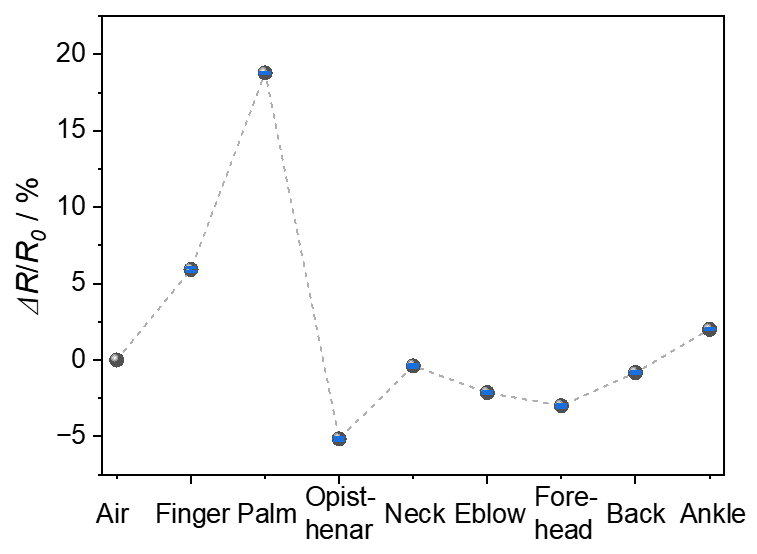


**Fig. S26** Normalized resistance change of different parts of the human body recorded by GT**2**


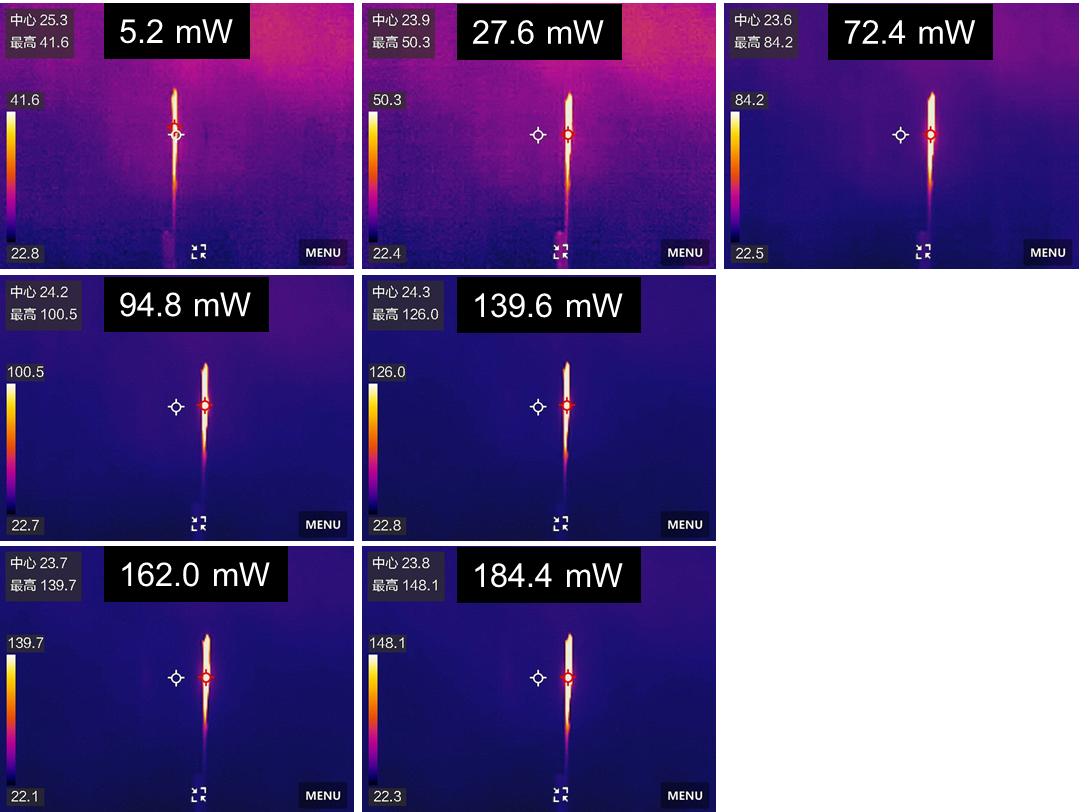


**Fig. S27** Infrared thermal imaging of GT**2** under varying infrared (808 nm) power intensities. Thermal images show the temperature distribution of GT**2** as the infrared power increases from 5.2 to 184.4 mW. The red circle indicates the maximum temperature reached on the crystal surface


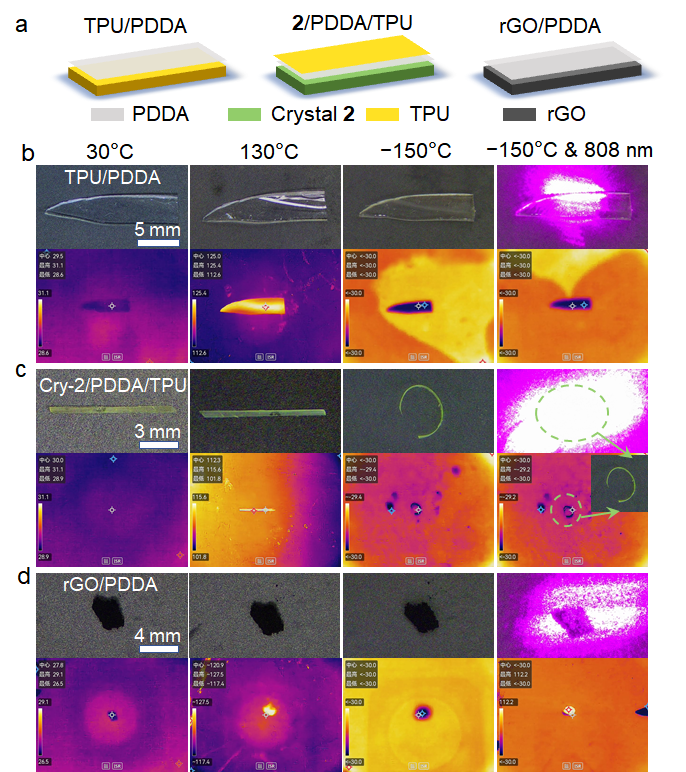


**Fig. S28** (**a**) Schematic illustration of the sample configurations: TPU/PDDA, **2**/PDDA/TPU, and rGO/PDDA. (**b–d**) Optical images (top) and infrared thermal images (bottom) of the samples TPU/PDDA (**b**), **2**/PDDA/TPU (**c**), and rGO/PDDA (**d**) under different temperatures and under low-temperature infrared irradiation


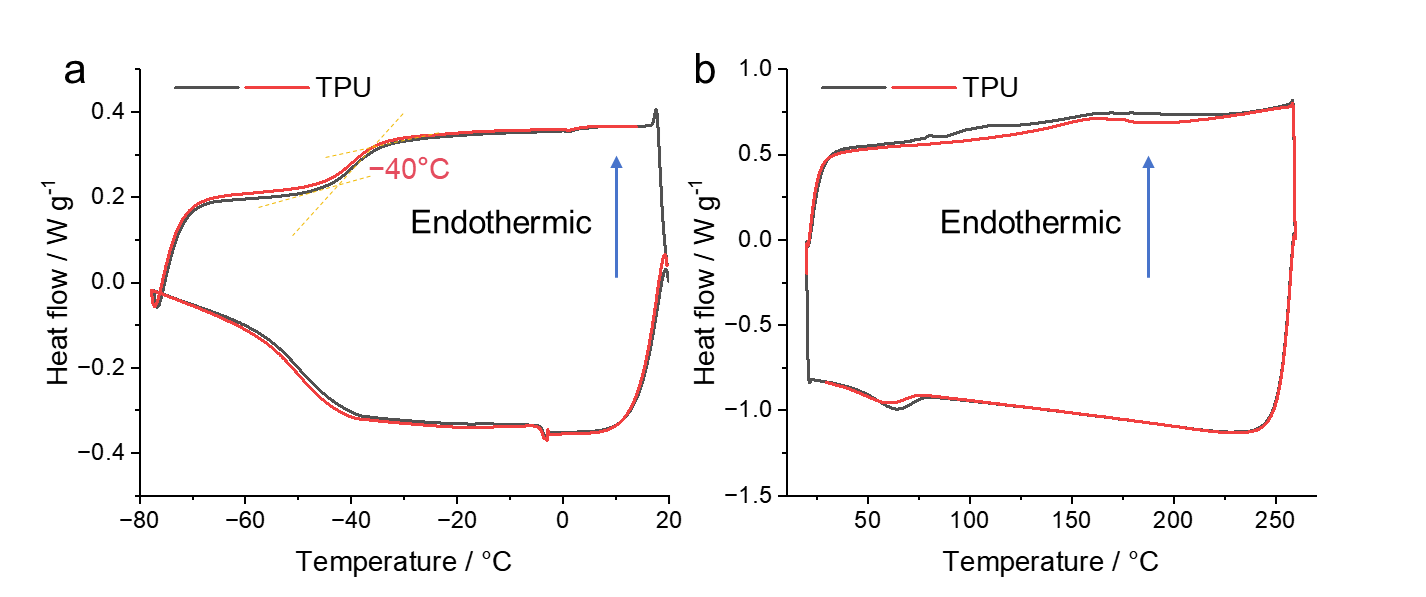


**Fig. S29** DSC curves of TPU in the ranges of 20 to −80°C and 20 to 260°C


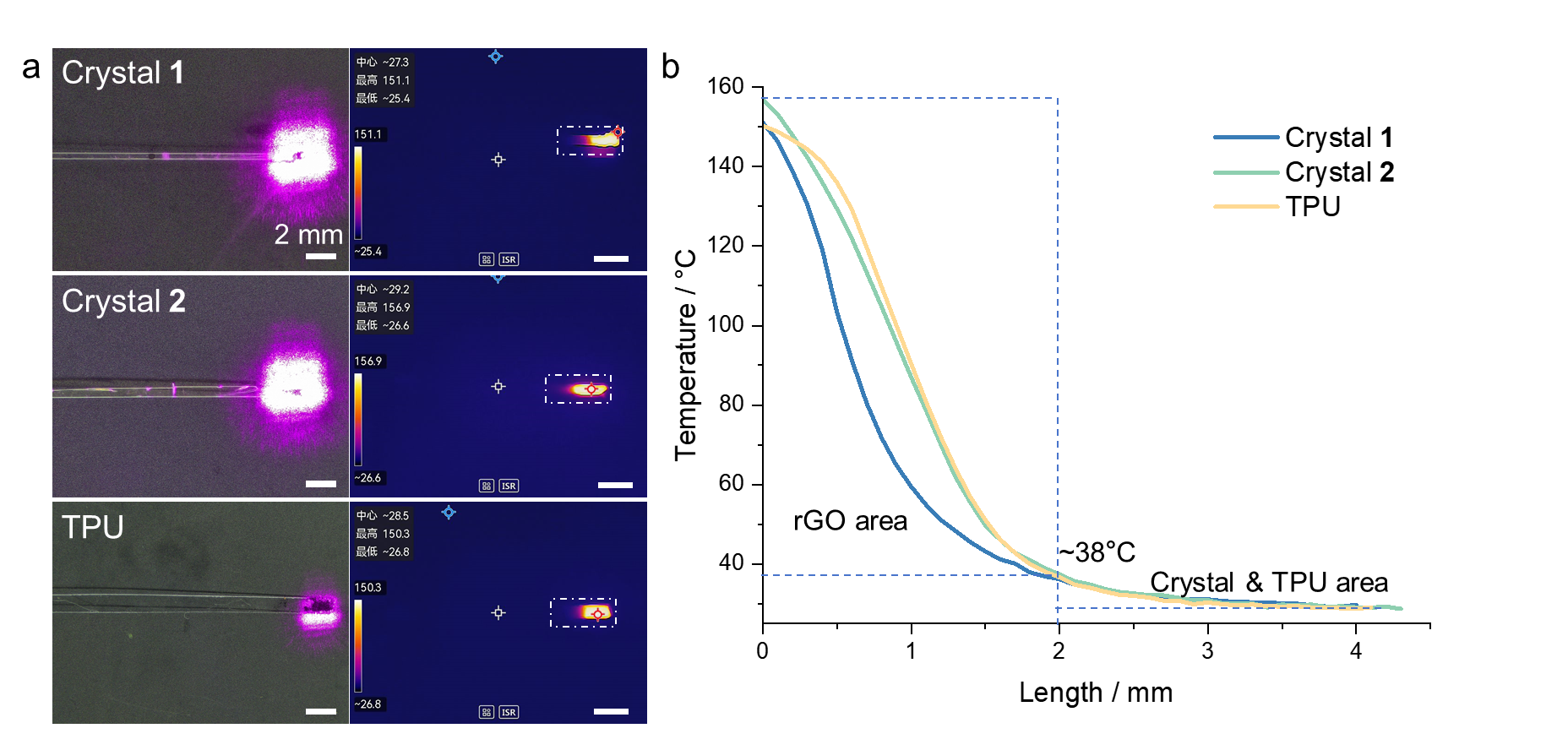


**Fig. S30** (**a**) Comparison of the thermal conductivity of Crystal **1**, Crystal **2**, and TPU. A ~2 mm area of rGO was attached to one end of each sample, and the region was irradiated with infrared light to raise its central temperature above 150°C. (**b**) The corresponding temperature distribution profiles along the white dashed box in panel (a)


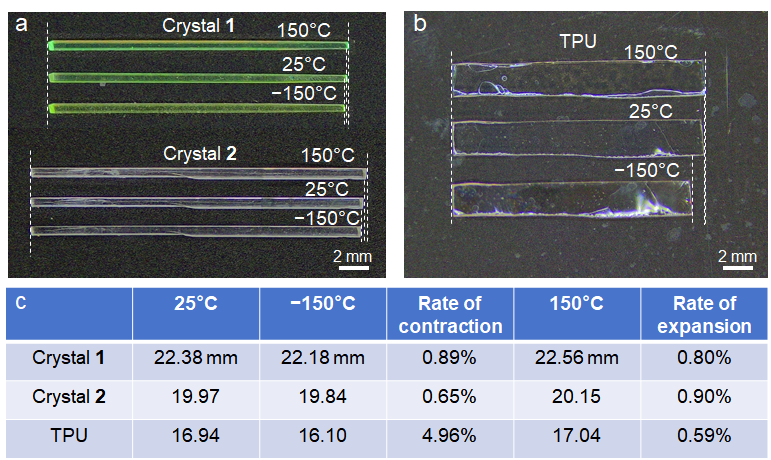


**Fig. S31** (**a, b**) Optical photographs of crystal **1** and **2** (**a**) and TPU film (**b**) at 25°C, 150°C and −150°C. (**c**) Corresponding length measurements with calculated contraction ratios (low temperature) and expansion ratios (high temperature)


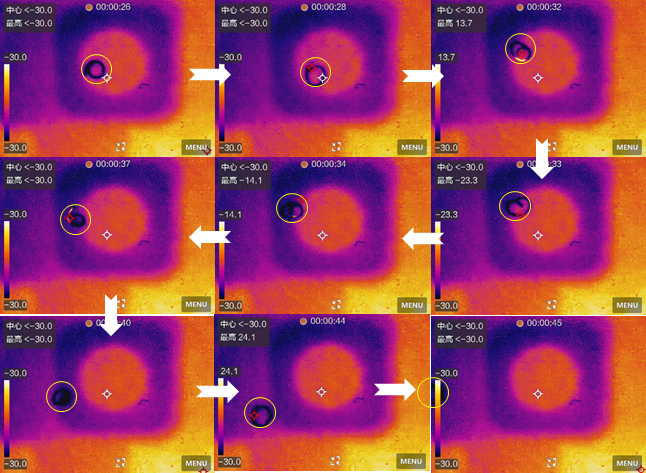


**Fig. S32** The motion and temperature changes of the crystal driven by infrared irradiation at low temperatures were captured using an infrared camera. The crystal is highlighted within the yellow circle. The actual environmental temperature is −150°C, while the infrared camera's detection limit is −30°C


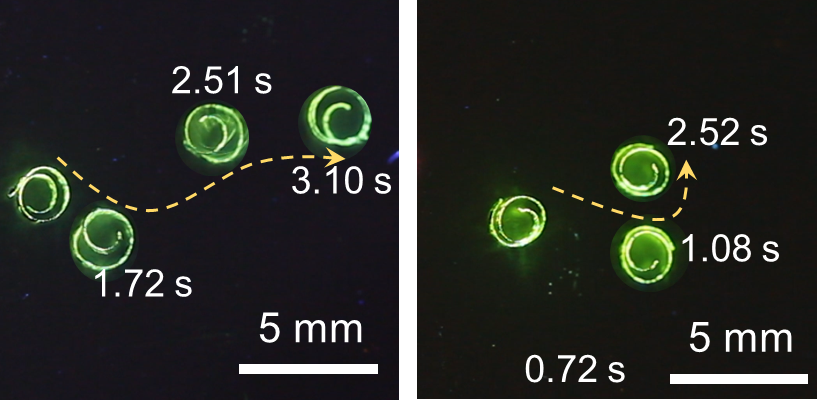


**Fig. S33** Fluorescent images showing the time and distance of curled GT**2** crystals at −150°C as it jumps under infrared driving. The dotted yellow lines represent the motion trajectory of the crystal


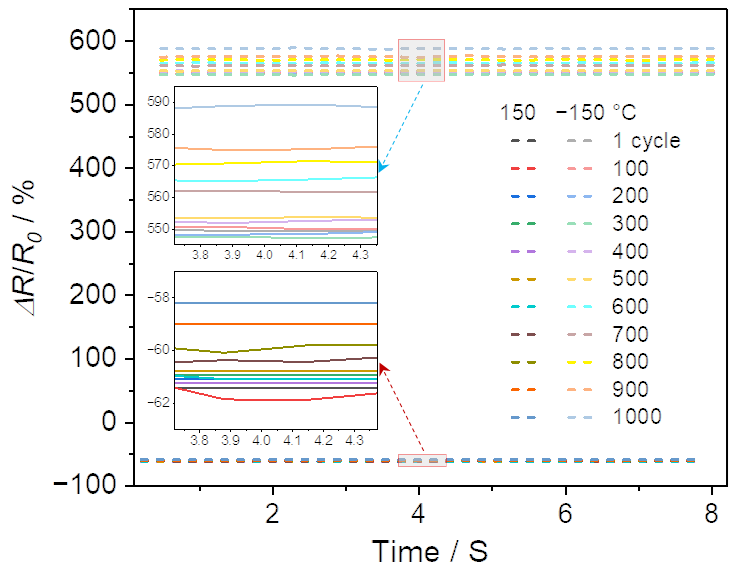


**Fig. S34** Fatigue resistance of GT**2** under thermal cycling. The *ΔR*/*R₀* of GT**2** over 1000 cycles between 150°C and −150°C demonstrates its excellent thermal stability and durability. Insets show magnified views at specific cycles


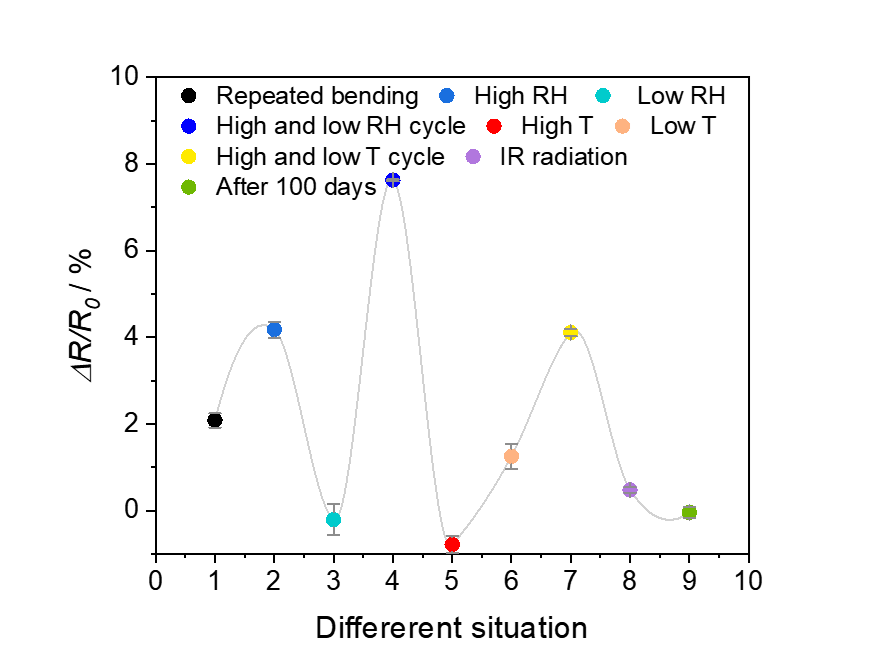


**Fig. S35** Normalized resistance changes of the GT**2** under different conditions


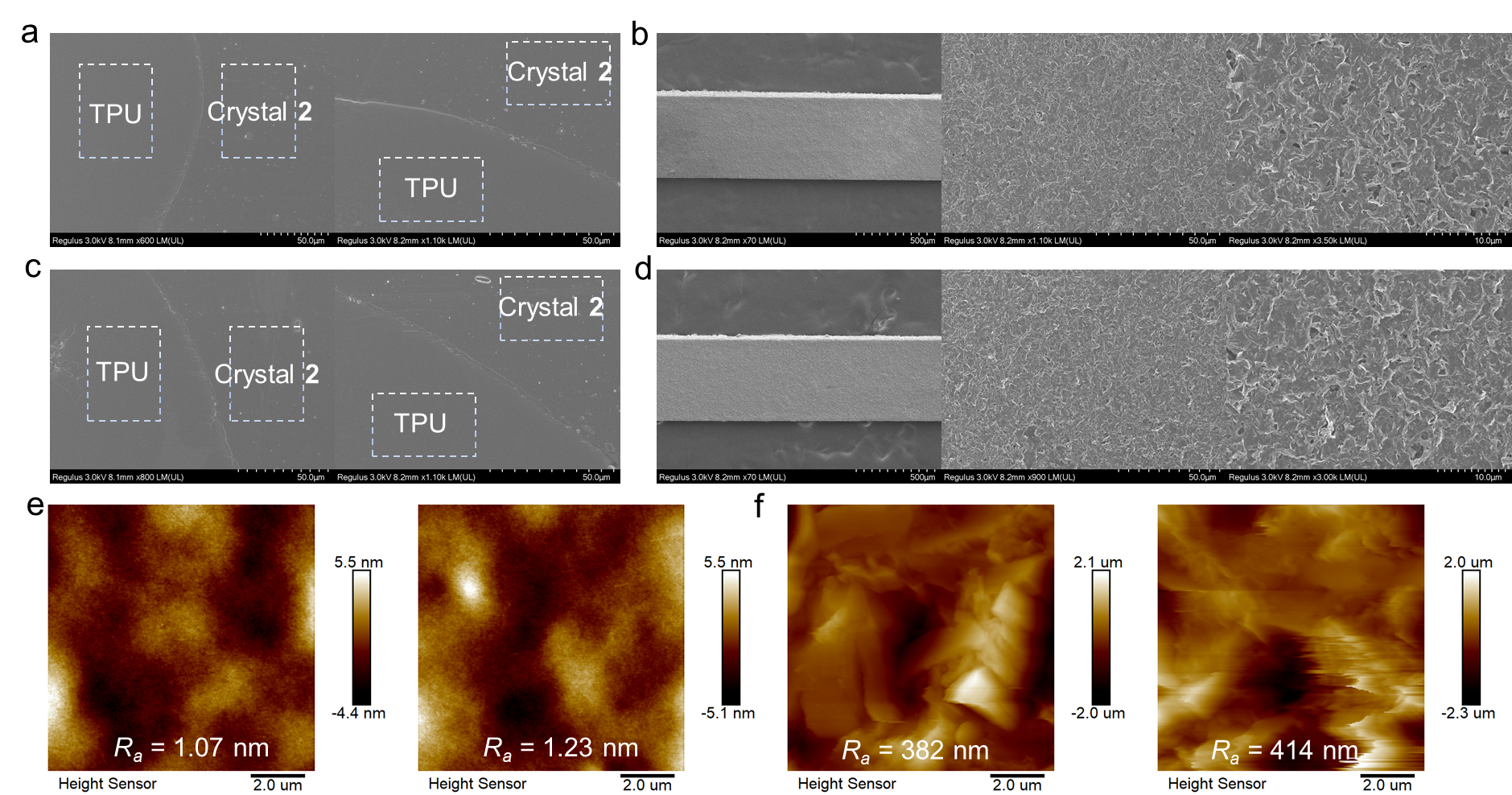


**Fig. S36** (**a, b**) Initial GT**2** showing the TPU and crystal surfaces (**a**) as well as the layered rGO structure (**b**). (**c, d**) GT**2** after cycles of high/low humidity (20%–90% RH), high/low temperature (−150 to 150°C), and repeated infrared irradiation, showing the TPU and crystal surfaces (**c**) together with the rGO layered structure (**d**). (**e**) AFM images of the TPU layer in GT**2** before (left) and after fatigue cycling (right). (**f**) AFM images of the rGO layer in GT**2** before (left) and after fatigue cycling (right).

**Table S1** Dimensions, plastic yield strength, and elastic modulus of crystals **1** and **2**, as well as hybrid crystals GT**1** and GT**2** in three-point bending tests

| Crystals | Sample number | Width / μm | Thickness / μm | Yield strength / Mpa | Elastic moduli / Gpa |
| --- | --- | --- | --- | --- | --- |
| **1** | 1 | 354.97 | 163.74 | 7.34 | 1.97 |
|  | 2 | 347.94 | 149.86 | 7.71 | 1.94 |
|  | 3 | 272.57 | 138.64 | 8.44 | 1.82 |
| GT**1** | 4 | 774.89 | 146.72 | 7.82 | 1.53 |
|  | 5 | 775.22 | 152.79 | 8.54 | 1.66 |
|  | 6 | 773.94 | 152.41 | 7.63 | 1.43 |
| **2** | 1 | 550.68 | 169.30 | 21.09 | 1.74 |
|  | 2 | 551.06 | 169.39 | 19.02 | 1.93 |
|  | 3 | 551.06 | 169.99 | 21.08 | 1.88 |
| GT**2** | 4 | 390.28 | 158.90 | 19.42 | 1.11 |
|  | 5 | 382.53 | 156.89 | 19.68 | 1.40 |
|  | 6 | 382.53 | 157.45 | 22.68 | 1.53 |

**Table S2** Dimensions, breaking strength, and tensile modulus of crystals **1** and **2**, as well as hybrid crystals GT**1** and GT**2** in tensile tests

| Crystals | Sample number | Length / mm | Width / μm | Thickness / μm | Yield strength / Mpa | Elastic modulus / Gpa |
| --- | --- | --- | --- | --- | --- | --- |
| **1** | 1 | 4.22 | 521 | 81 | 5.10 | 1.11 |
|  | 2 | 5.68 | 427 | 84 | 6.13 | 1.19 |
|  | 3 | 6.77 | 496 | 102 | 7.54 | 1.37 |
| GT**1** | 4 | 8.1 | 608 | 150 | 5.67 | 1.09 |
|  | 5 | 5.07 | 384 | 152 | 5.97 | 1.24 |
|  | 6 | 6.17 | 496 | 96 | 7.61 | 1.31 |
| **2** | 1 | 2.89 | 426 | 117 | 5.70 | 0.645 |
|  | 2 | 5.19 | 263 | 104 | 5.96 | 0.695 |
|  | 3 | 3.02 | 272 | 94 | 4.36 | 0.624 |
| GT**2** | 4 | 3.56 | 319 | 122 | 4.59 | 0.690 |
|  | 5 | 2.78 | 282 | 120 | 4.49 | 0.679 |
|  | 6 | 2.69 | 257 | 175 | 6.17 | 0.700 |

**Table S3** Comparison of the performance of our hybrid crystal-based flexible sensor with recently reported flexible sensors. The comparison parameters include operating humidity range (RH / %), operating temperature range (T / °C), sensitivity （*S*） and detection limit (*MDL*). “―” indicates no data available

| Sensor system | Humidity sensing | | | Temperature sensing | | |  |
| --- | --- | --- | --- | --- | --- | --- | --- |
| Parameters | RH / % | *S* / % RH^–1^ | *MDL /* % RH | T / °C | *S* / % °C^–1^ | *MDL /* °C | Refs. |
| This work (hybrid crystal) | 25–90 | 1.65 | 0.54 | 25–180 | 0.46 | 1.77 |  |
| AgNPs/CA/PVA | 50–90 | ― | ― | 30–45 | 0.043 | ― | [S1] |
| Dual-modal sensors | 20–90 | ― | ― | 25–45 | 1.66 | ― | [S2] |
| FET-based thermistor | ― | ― | ― | 30–80 | 1.34 | 0.2 | [S3] |
| RF/PAA DN gel | 70–90 | 0.311 | ― | −20–90 | 8.65 | ― | [S4] |
| PVA/CA/AgNPs sensor | 30–90 | ― | ― | 30–40 | 0.076 | ― | [S5] |
| Biomimetic electronic skins | 33–98 | ― | ― | 25–50 | 1.74 | ― | [S6] |
| PVA-CNF organohydrogel. | 22–98 | ― | ― | 21.5–48.6 | 9.38 | ― | [S7] |
| PPy/PU-CNT nano-architectures | 20–80 | 3.71 | ― | 20–50 | 0.77 | ― | [S8] |
| Self-powered multimodal sensor | 40–90 | ― | ― | 25-40 | 0.055 | ― | [S9] |

**Table S4.** Comparison of cryogenic actuation performance of our hybrid crystals with representative flexible actuators

| Actuator system | Actuation mode | Operating temperature | Motion Type | Unique advantage | Refs. |
| --- | --- | --- | --- | --- | --- |
| This work  (hybrid crystal) | Infrared radiation | −150°C | Crawl, walk, jump | Fast and controllable movement |  |
| IS-P^3^//1–3 | Temperature switching | −125°C | Bending,  grasping | Sensitive response (1867°/s) | [S10] |
| Magnetic flexible metastructures | Magnetic control | −196°C | Crawling, grasping | Complex deformation | [S11] |
| LCEs | Temperature switching | −18°C | Slight bending | Simple fabrication | [S12] |

**S2** **Legends for the Supplementary Movies**

**Movie S1** Infrared thermographic imaging of infrared-driven heating and natural cooling of the hybrid crystal GT**2**. The movie captures the temperature evolution of GT**2** under four different infrared power levels, showing rapid heating to peak temperatures followed by natural cooling after the infrared source is turned off.

**Movie S2** Low-temperature-driven response of GT**2**. The movie shows the crystal curling into a spring-like shape when placed near liquid nitrogen and returning to its original straight form upon exposure to room temperature, demonstrating its reversible low-temperature actuation.

**Movie S3** Infrared thermographic imaging of shape transformation and localized temperature changes of the curled crystal at low temperatures. At low temperatures, the crystal curls into a helical shape, and upon infrared irradiation at a specific site, localized heating induces partial straightening, triggering significant shape transformation and motion. This reversible process demonstrates the crystal's photothermal responsiveness.

**Movie S4** Infrared-driven inching and helical motions of bent GT**3** and curled GT**2** crystals at low temperatures.

**Movie S5** Infrared-driven jumping and obstacle-crossing motion of GT**2** at low temperatures.

**Movie S6** Infrared-driven walking motion of GT**4** with paper feet at low temperatures, demonstrating controlled directional movement upon periodic infrared irradiation.

**Supplementary References**

1. L. Chen, Y. Xu, Y. Liu, J. Wang, J. Chen et al., Flexible and transparent electronic skin sensor with sensing capabilities for pressure, temperature, and humidity. ACS Appl. Mater. Interfaces **15**, 24923–24932 (2023). <https://doi.org/10.1021/acsami.3c03829>
2. W. Shi, X. Yang, L. Lei, J. Lin, Q. Liang et al., Human respiration monitoring using humidity and temperature dual-modal sensors for temperature-insensitive humidity sensing and synchronous temperature sensing. Sens. Actuators A Phys. **395**, 117008 (2025). <https://doi.org/10.1016/j.sna.2025.117008>
3. T.Q. Trung, S. Ramasundaram, B.U. Hwang, N.E. Lee, An all-elastomeric transparent and stretchable temperature sensor for body-attachable wearable electronics, Adv. Mater. **28**, 502–509 (2016). <https://doi.org/10.1002/adma.201504441>
4. Q. Quan, C. Fan, N. Pan, M. Zhu, T. Zhang et al., Tough and stretchable phenolic-reinforced double network deep eutectic solvent gels for multifunctional sensors with environmental adaptability. Adv. Funct. Mater. **33**, 2303381 (2023). <https://doi.org/10.1002/adfm.202303381>
5. L. Chen, X. Chang, H. Wang, J. Chen, Y. Zhu. Stretchable and transparent multimodal electronic-skin sensors in detecting strain, temperature, and humidity, Nano Energy **96**, 107077 (2022). <https://doi.org/10.1016/j.nanoen.2022.107077>
6. W. Wang, D. Yao, H. Wang, Q. Ding, Y. Luo et al., A breathable, stretchable, and self-calibrated multimodal electronic skin based on hydrogel microstructures for wireless wearables. Adv. Funct. Mater. **34**, 2316339 (2024). <https://doi.org/10.1002/adfm.202316339>
7. J. Li, H. Wang, Y. Luo, Z. Zhou, H. Zhang, Design of AI-enhanced and hardware-supported multimodal e-skin for environmental object recognition and wireless toxic gas alarm. Nano-Micro Lett. **16**, 256 (2024). <https://doi.org/10.1007/s40820-024-01466-6>
8. Y. He, S. Xiao, Q. Wang, C. Guo, D. Luo et al., An in-situ multiresponsive flexible sensor utilizing electrochemical impedance spectroscopy for the precise and simultaneous detection of pressure, temperature, and humidity. Chem. Engin. J. **521**, 166956 (2025). <https://doi.org/10.1016/j.cej.2025.166956>
9. L. Ye, Z. Tang, J. Feng, Y.-P. Jiang, X.-G. Tang et al., A flexible self-powered multimodal sensor with low-coupling temperature, pressure and humidity detection for physiological monitoring and human-robot collaboration. Chem. Eng. J. **519**, 164866 (2025). <https://doi.org/10.1016/j.cej.2025.164866>
10. L. Lan, L. Li, Q. Di, X. Yang, X. Liu et al., Organic single-crystal actuators and waveguides that operate at low temperatures. Adv. Mater. **34**, 2200471 (2022). <https://doi.org/10.1002/adma.202200471>
11. W. Han, W. Gao, X. Wang. Magnetic flexible metastructures: developing smart shape-morphing capabilities at extremely low temperatures. Compos. Sci. Technol. **269**, 111242 (2025). <https://doi.org/10.1016/j.compscitech.2025.111242>
12. Y. Chen, X. Li, J. Wang, D. Zhou, L. Yao et al., Ultra-low temperature-responsive liquid crystal elastomers with tunable drive temperature range. Polymer **294**, 126726 (2024). <https://doi.org/10.1016/j.polymer.2024.126726>
